# Astrocyte-Derived Thrombospondin Induces Cortical Synaptogenesis in a Sex-Specific Manner

**DOI:** 10.1523/ENEURO.0014-21.2021

**Published:** 2021-07-28

**Authors:** Anna Mazur, Ean H. Bills, Kayla M. DeSchepper, James C. Williamson, Brandon J. Henderson, W. Christopher Risher

**Affiliations:** Department of Biomedical Sciences, Joan C. Edwards School of Medicine at Marshall University, Huntington, WV 25701

**Keywords:** astrocytes, cortex, estrogen, sex differences, synaptogenesis, thrombospondin

## Abstract

The regulation of synaptic connectivity in the brain is vital to proper functioning and development of the CNS. Formation of neural networks in the CNS has been shown to be heavily influenced by astrocytes, which secrete factors, including thrombospondin (TSP) family proteins, that promote synaptogenesis. However, whether this process is different between males and females has not been thoroughly investigated. In this study, we found that cortical neurons purified from newborn male rats showed a significantly more robust synaptogenic response compared with female-derived cells when exposed to factors secreted from astrocytes. This difference was driven largely by the neuronal response to TSP2, which increased synapses in male neurons while showing no effect on female neurons. Blockade of endogenous 17β-estradiol (E2) production with letrozole normalized the TSP response between male and female cells, indicating a level of regulation by estrogen signaling. Our results suggest that male and female neurons show a divergent response to TSP synaptogenic signaling, contributing to sex differences in astrocyte-mediated synaptic connectivity.

## Significance Statement

The regulation of synaptic connectivity by astrocytes has been a focus of both the neurodevelopmental and glial biology fields for nearly two decades, but many key findings did not take into account the possibility of sex differences. For the first time, we show that a prominent astrocyte signaling factor, thrombospondin-2 (TSP2), strongly promotes synapse formation in male but not female neurons. Furthermore, this sex difference can be abolished by inhibiting production of 17β-estradiol (E2), revealing that this astrocyte synaptogenic pathway is regulated by estrogen. Our findings should prompt serious consideration into the ramifications of sex and sex hormones on astrocyte synaptic signaling as well as encourage and inform future studies, including potential implications for neurodevelopmental disorders (NDDs) that present with strong sex biases.

## Introduction

Neurons form complex arrangements throughout the CNS that form the basis of our ability to think, move, learn, and remember. Synapses, the basic functional units of the CNS, represent the coming together of an axon from a presynaptic cell and a dendrite from a postsynaptic one. Precise timing and location of synapse formation (i.e., synaptogenesis) is critical for the function of the developing brain, and it has become increasingly apparent that astrocytes are powerful regulators of this process (Barres, 2008). The last 20+ years have seen an explosion of discovery of astrocytic mechanisms that influence synaptogenesis, both positively and negatively, via both contact-mediated signaling as well as secreted factors (Allen and Eroglu, 2017). However, despite this greatly increased awareness, a significant caveat of most of these studies was the lack of consideration for potential sex differences in astrocyte-mediated synaptic development.

The brains of males and females show significant structural, chemical, and functional differences (Pfaff and Joëls, 2017). This phenomenon holds true over the course of “normal” development as well as in cases of aberrant CNS function, such as that seen in neurodevelopmental and neurodegenerative disorders (Hanamsagar and Bilbo, 2016). The release of steroids from the gonads beginning with puberty certainly plays a major role in defining sex differences in the still-maturing brain (Genazzani et al., 2007; Ho et al., 2020), but relatively little is known about how prepubertal cellular processes may influence neural circuits in a distinct, sex-dependent manner. Many previous investigations into astrocyte-induced synaptogenesis were performed on neurons isolated from mixed-sex litters of rats and mice, often using astrocyte-conditioned media (ACM) or astrocyte culture inserts which were similarly generated from mixed-sex sources (Allen et al., 2012; Christopherson et al., 2005; Eroglu et al., 2009; Hughes et al., 2010; Kucukdereli et al., 2011; Farhy-Tselnicker et al., 2017; Blanco-Suarez et al., 2018).

As a novel approach, for this study, we used cells isolated specifically from either males or females to identify sex differences in astrocyte-mediated synapse formation. Neuronal cultures isolated from the cortices of male rats experienced a significantly greater fold increase in excitatory synapses when exposed to astrocyte-secreted factors than did cultures purified from females. Driving this sex difference was the strongly synaptogenic response of male-derived neurons to the matricellular protein thrombospondin-2 (TSP2), which was not observed in female cultures. Intriguingly, our data suggest that the divergence may be, at least in part, because of transient differences in *de novo* neuronal estrogen synthesis during the peak synaptogenic window for astrocytes and TSP.

## Materials and Methods

### Animals

All animal procedures were performed in accordance with the Marshall University Institutional Animal Care and Use Committee’s regulations. Adult Sprague Dawley rat mothers and their mixed sex newborn litters were obtained from Hilltop Lab Animals. Following sex determination (based on visual examination of the anogenital region), pups were separated into male and female before dissection and cell purification.

### Cortical neuron purification and treatment

Cortical neurons were purified separately from male and female postnatal day (P)1 Sprague Dawley rats by sequential immunopanning. Briefly, following rapid decapitation, brains were removed and cortices were dissected. Following 45 min of digestion in papain (∼7.5 units/ml; Worthington) supplemented with DNase (Worthington) dissolved in Dulbecco’s PBS (DPBS; Invitrogen) at 34°C, sequential low/high concentrations of ovomucoid inhibitor (Worthington) was used to gradually halt papain activity. The cell solution was then passaged through a 20-μm Nitex mesh filter (Sefar) before a series of negative immunopanning steps. Petri dishes coated with Bandeiraea Simplicifolia Lectin I (Vector Laboratories), followed by AffiniPure goat-anti mouse IgG+IgM (H + L; Jackson ImmunoResearch) and AffiniPure goat-anti rat IgG+IgM (H + L; Jackson ImmunoResearch) antibodies were used to remove non-neuronal cells and general debris. To further purify neurons (>95%) using positive immunopanning, the cell solution was passaged onto Petri dishes coated with mouse antibody against neural cell adhesion molecule L1 (ASCS4; Developmental Studies Hybridoma Bank, University of Iowa). Following final washes, pelleting and resuspension in serum-free NGM [Neurobasal (Invitrogen), B27 supplement (Invitrogen), 2 mm GlutaMax (Invitrogen), 100 U/ml pen/strep (Invitrogen), 1 mm NaPyruvate (Invitrogen), 50 ng/ml BDNF (Peprotech), 20 ng/ml CNTF (Peprotech), 4.2 μg/ml forskolin (Sigma), and 10 μm AraC (Sigma)], neurons were plated at a density of 60K/well on poly-D-lysine (PDL; Sigma) and laminin (R&D Systems)-coated coverslips in a 24-well plate. After 2 d *in vitro* (DIV2) at 37°C/5% CO_2_, half of the NGM in each well was replaced with fresh NGM of the same composition with the exception of: Neurobasal Plus (Invitrogen) instead of Neurobasal and GlutaMax, B27 Plus (Invitrogen) instead of B27, and no AraC; this media was then used for feedings every 2–3 d for the duration of the experiment before electrophysiology or fixation for synaptic immunocytochemistry (ICC) on DIV13. Recombinant TSP2 protein was purified from CHO cells expressing mouse TSP2 via affinity chromatography with HiTRAP heparin HP (GE Healthcare). Standard TSP2 treatment of neurons was performed on DIV7 and DIV10 at a dose of 500 ng/ml.

### Cortical astrocyte isolation, culture, and ACM harvesting

Cortical astrocytes were purified separately from male and female P1 Sprague Dawley rats following a similar protocol to neurons, as described above. After the Nitex mesh filtering step, the cells were pelleted and resuspended in astrocyte growth media [AGM; DMEM + GlutaMax (Invitrogen), 10% heat-inactivated FBS (Sigma), 10 μm hydrocortisone (Sigma), 100 U/ml pen/strep, 5 μg/ml insulin (Sigma), 1 mm NaPyruvate, 5 μg/ml N-acetyl-L-cysteine (NAC; Sigma)]. After counting, 15–20 million cells were plated on PDL-coated 75-mm^2^ flasks and incubated at 37°C/5% CO_2_. On DIV3, AGM was removed and replaced with DPBS. In order to isolate the adherent monolayer of astrocytes, flasks were shaken vigorously by hand 3–6× for 15 s each. DPBS was then replaced with fresh AGM. AraC was added to the AGM from DIV5–DIV7 to minimize astrocyte proliferation. On DIV7, astrocytes were passaged into either transwell inserts (Corning) at a density of 125K (for direct culture with neurons) or at 3 million per 100-mm tissue culture dish (for the generation of ACM).

For the generation and collection of ACM, at 24 h postpassaging into 100-mm dishes, astrocytes were washed 3× with DPBS and conditioned at 37°C/5% CO_2_ without disturbing for 4 d in minimal medium [Neurobasal (phenol red-free), 2 mm L-g(Invitrogen), 1 mm NaPyruvate, 100 U/ml pen/strep, 5 μg/ml NAC, 40 ng/ml T3 (Sigma), and Sato supplement (Winzeler and Wang, 2013)]. After conditioning, media was collected from dishes and centrifuged to pellet cell debris before concentrating in 5000 M.W.C.O. Vivaspin tubes (Sartorius). ACM was then aliquoted and flash frozen in liquid nitrogen until use. Standard ACM treatment of neurons was performed on DIV7 and DIV10 at a dose of 75 μg/ml before fixation for synaptic ICC on DIV13.

### Synaptic ICC

DIV13 neurons in 24-well plates were fixed with warm 4% paraformaldehyde (PFA; Electron Microscopy Sciences) in PBS for 7 min. Next, PFA was removed and cells were washed 3× with PBS then blocked in a buffer containing 0.2% Triton X-100 (Roche) in 50% normal goat serum (NGS; Jackson ImmunoResearch)/50% antibody buffer (PBS containing 1% BSA, 0.04% NaN3, 0.2% Triton X-100) at room temperature (RT) for 30 min. Following another 3× PBS wash, cells were treated with 10% NGS/90% antibody buffer containing primary antibodies against Bassoon (1:500; mouse; Enzo/Assay Designs) and Homer1 (1:500; rabbit; Synaptic Systems), at 4°C overnight in the dark. The next morning, another 3× PBS wash was performed, followed by a 2-h RT incubation in 10% NGS/90% antibody buffer containing the following fluorescently-conjugated secondary antibodies: goat anti-mouse Alexa Fluor 488 (1:500; Invitrogen) and goat anti-rabbit Alexa Fluor 594 (1:500; Invitrogen). After final round of 3× PBS washes, coverslips were transferred to glass slides with Vectashield mounting medium containing DAPI (Vector Laboratories), sealed with nail polish, and imaged on a Leica DM5500B microscope with a 63×/1.4 NA objective at 1920 × 1440 resolution.

### Whole-cell patch clamp electrophysiology

Glass coverslips with neuronal cultures were detached from imaging dishes and placed in the recording chamber of an upright microscope (Axio Examiner, Zeiss) while being continually perfused by extracellular solution (ECS; 140 mm NaCl, 5 mm KCl, 2 mm CaCl_2_, 1 mm MgCl_2_, 10 mm HEPES, and 10 mm glucose). Electrophysiological signals were recorded from visually identified neurons with a Sutter IPA and SutterPatch software. Patch pipettes were filled with solution containing the following: 135 mm K gluconate, 5 mm KCl, 5 mm EGTA, 0.5 mm CaCl_2_, 10 mm HEPES, 2 mm Mg-ATP, and mm 0.1 GTP (pH was adjusted to 7.2 with Tris-base, and osmolarity was adjusted to 280–300 mOsm with sucrose). The resistance of patch pipettes was 4–8 MΩ. Junction potential was nulled just before forming a gigaseal. Series resistance was monitored without compensation throughout the experiment. Data were discarded if the series resistance (10–25 MΩ) changed by >20% during recordings. All recordings were done at RT. After achieving a gigaseal, gentle suction was used to achieve whole cell configuration. The neuron was voltage clamped at −70 mV to record miniature EPSCs (mEPSCs) in the presence of 1.0 μm tetrodotoxin. Data were sampled at 10 kHz and filtered at 2 kHz.

### Western blotting

Western blotting was performed on media collected from DIV10 cultured astrocytes or from cortical neuron lysates prepared on DIV13. For lysate preparation, NGM was aspirated and cells washed twice with TBS (+ Mg^2+^ + Ca^2+^) and lysed in ice-cold solubilization buffer (25 mm Tris, pH 7.4, 150 mm NaCl, 1 mm CaCl_2_, and 1 mm MgCl_2_) containing 0.5% NP-40 (Thermo Fisher Scientific) and EDTA-free protease inhibitors (Roche). Cells were spun at max speed at 4°C for 10 min and supernatant was collected. The protein concentrations of the lysates were determined using micro-BCA protein assay kit (Pierce); 20-μg protein was loaded into each well. Samples were resolved by SDS-PAGE on 4–15% stain-free polyacrylamide gels (Bio-Rad) and transferred onto a methanol-activated Immobilon-FL PVDF membrane (EMD Millipore). Before blocking, blot was imaged for total protein content on Bio-Rad Chemidoc MP for normalization purposes. Blots were blocked in 50% fluorescent blocking buffer in PBS (MB-070; Rockland) for 1 h at RT. Blots were incubated with primary antibody dilution in blocking buffer plus 0.1% Tween 20: goat anti-TSP2 (AF1635; R&D Systems); mouse anti-DHP receptor, α2 subunit, 1:500 (D219; Sigma-Aldrich) overnight at 4°C. Fluorescently labeled secondary antibodies (Multifluor Red and Green, ProteinSimple) were diluted (1:1000) in the same buffer as primary antibodies, and Western blottings were incubated with secondary antibodies for 1 h at RT in the dark. Detection was performed using the Bio-Rad Chemidoc MP. Quantification of band intensity was performed with Image Lab software (Bio-Rad) using the imaged total protein blot for normalization.

### ELISA for estradiol

Cortical neurons isolated separately from male and female P1 Sprague Dawley rats were plated at 60K/well into 48-well plates coated with PDL and laminin as described previously. Astrocytes were isolated from the same litter as described above, then plated at 600K/well into six-well plates on DIV7. Starting on DIV1 (neurons) or DIV5 (astrocytes), medium was collected daily, two wells pooled and flash-frozen per time point, until DIV12. Concentration of estradiol in collected medium was then determined with Parameter Estradiol assay (R&D Systems) according to instructions provided by the manufacturer. Briefly, medium was thawed on ice, centrifuged to precipitate cellular debris, and incubated in ELISA plate wells coated with primary antibody against estradiol, in the presence of fixed amount of HRP-conjugated estradiol. Next, the absorbance was read at 450 nm and corrected by reading at 570 nm. Data were analyzed using the four-parameter logistic (4-PL) curve-fit, per manufacturer recommendations, generated with Prism software (GraphPad).

### Image analysis and statistics

Synapse quantification was performed using the Puncta Analyzer plugin (Ippolito and Eroglu, 2010) developed by B. Wark for ImageJ (NIH). Analysis was performed by a trained user who was blinded as to condition/treatment. The plugin allows for rapid counting of presynaptic (i.e., Bassoon), postsynaptic (i.e., Homer1), and co-localized synaptic puncta, determined by user-defined thresholds for each individual channel. This approach provides an accurate estimation of synapse number based on the precise localization of presynaptic and postsynaptic proteins, which are typically non-overlapping when imaged with fluorescence-based ICC except when directly opposed at synapses.

Briefly, the user begins by designating a circular region of interest (ROI) approximately one-cell diameter radially surrounding the neuronal soma. The plugin then separates raw images into “green” (488/Bassoon) and “red” (594/Homer1) channels, subtracts background (rolling ball radius = 50), and then prompts the analyst to define a threshold with the goal of isolating “true” puncta while keeping noise to a minimum (which is facilitated by setting a minimum pixel size value of 4 in the user-selectable settings). The plugin then displays a results window with numbers of presynaptic, postsynaptic, and co-localized synaptic puncta that can be copied to a Microsoft Excel spreadsheet.

**Figure 1.**
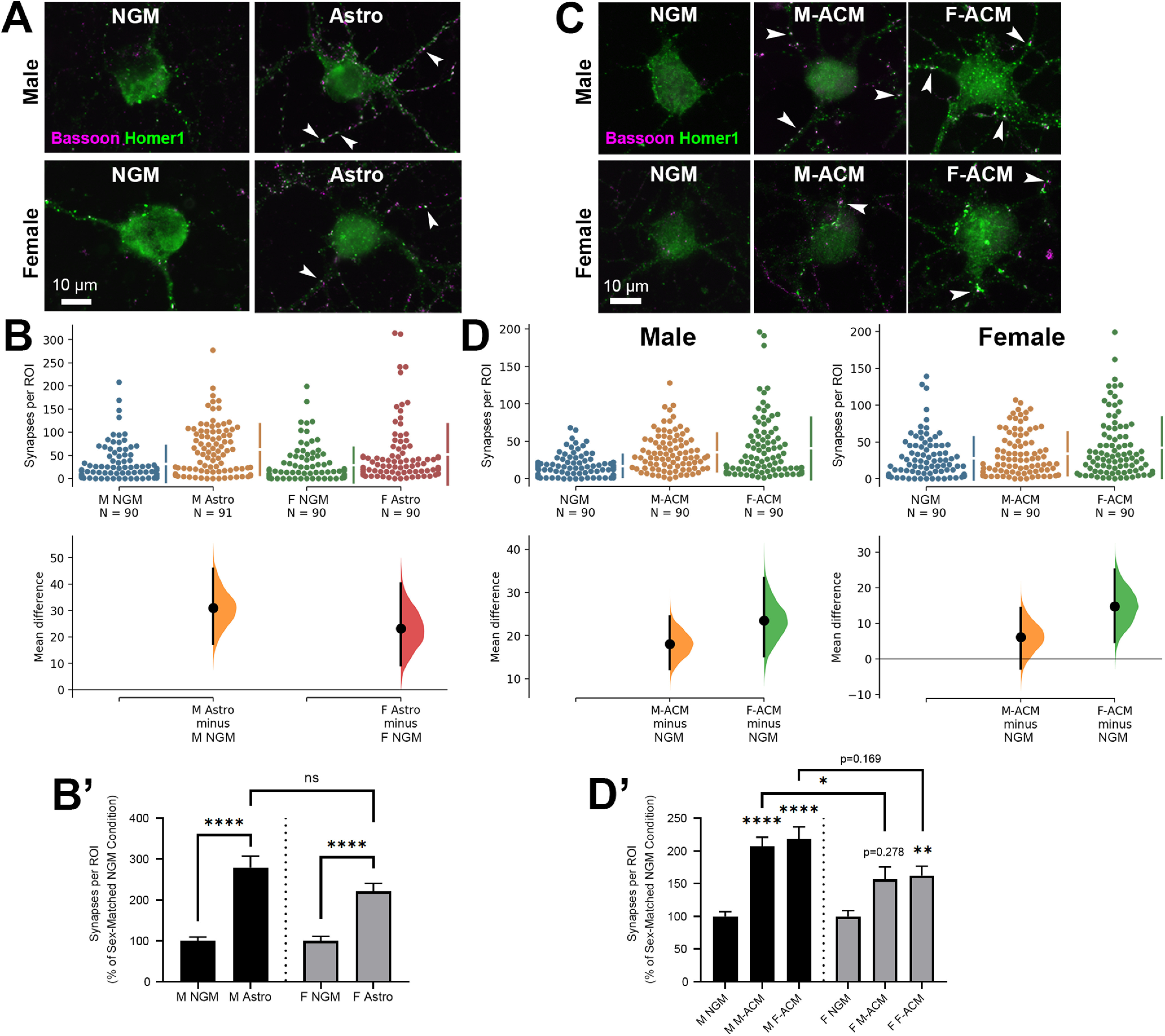
Sex differences in astrocyte-mediated synaptogenesis. ***A***, Representative ICC images of cortical neurons isolated from either male (top row) or female (bottom row) pups and treated with either NGM only or cultured with sex-matched astrocyte inserts (Astro) that allow for the continuous exchange of media/factors between cell types. Co-localized presynaptic (Bassoon; magenta) and postsynaptic (Homer1; green) puncta reveal sites of excitatory synapses (white arrowheads). ***B***, Exposure to astrocyte-secreted factors via culture inserts significantly increased excitatory synapse number per ROI in both sexes. ***B’***, Fold-change of synapse number induced by male or female-derived astrocyte inserts (shown as percent of sex-matched NGM control); ****p < 0.0001; n.s., not significant (Kruskal–Wallis test with Dunn’s multiple comparisons *post hoc* analysis; K-W statistic = 65.23). ***C*,** Representative ICC images of male (top row) and female (bottom row)-derived cortical neurons treated with ACM previously purified from either males or females (M-ACM and F-ACM, respectively), stained as in **A**. ***D***, ACM derived from either sex promoted excitatory synapses in male neurons. In females, this increase was limited to F-ACM. ***D’***, Fold-change of synapse number induced by male or female-derived ACM (shown as percent of sex-matched NGM control); *p < 0.05, **p < 0.01, ****p < 0.0001 (Kruskal–Wallis test with Dunn’s multiple comparisons *post hoc* analysis; K-W statistic = 77.03). In ***B***, ***D***, the mean difference for each comparison is shown in the Cumming estimation plot. The raw data are plotted on the upper axes; each mean difference (within sex) is plotted on the lower axes as a bootstrap sampling distribution. Mean differences are depicted as dots; 95% CIs are indicated by the ends of the vertical error bars.

**Figure 2.**
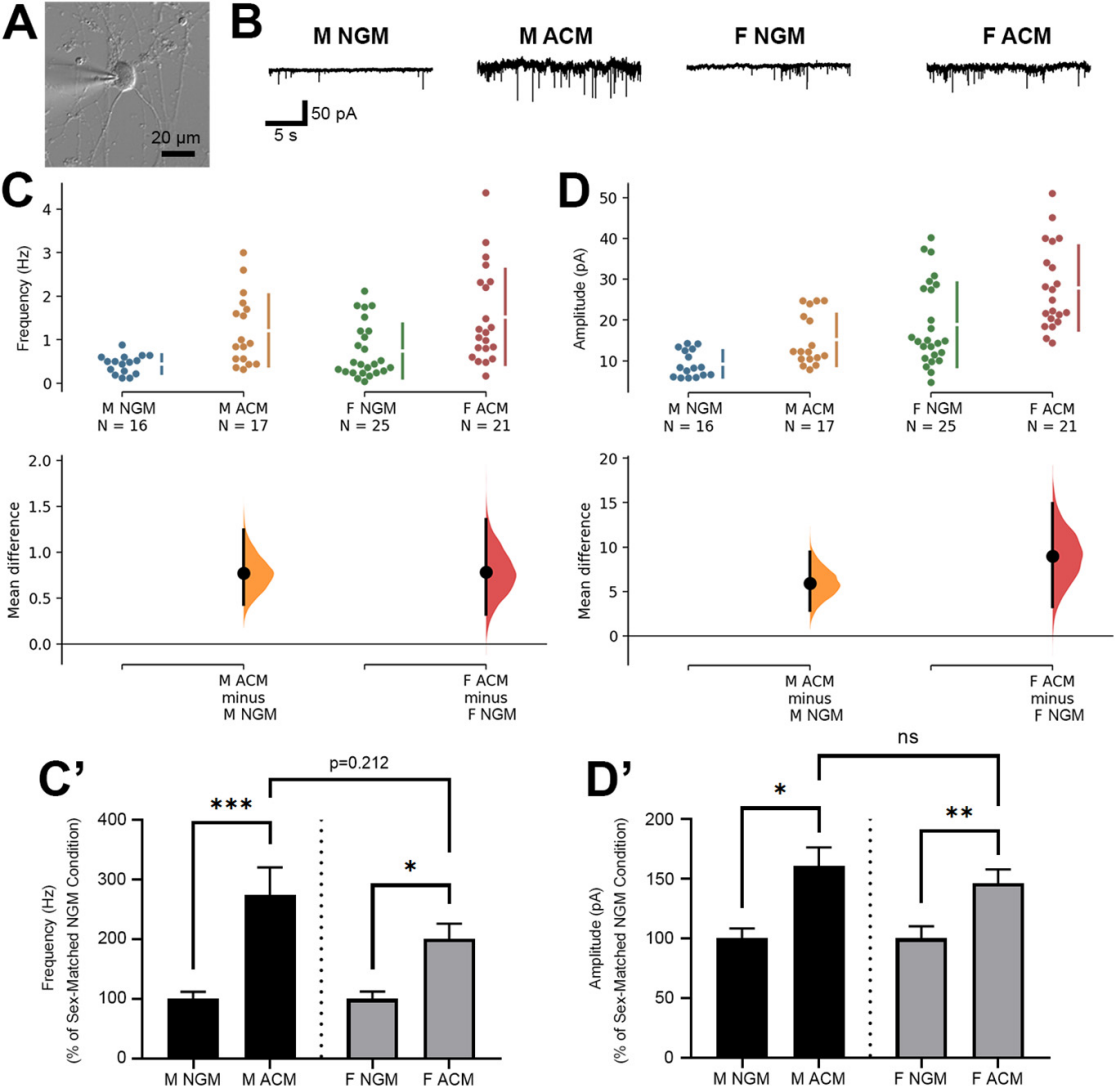
***A***, Brightfield image of a cortical neuron in whole cell patch clamp configuration for the recording of mEPSCs. ***B***, Sample mEPSC traces from male and female-derived cortical neurons treated with either standard NGM or NGM with the addition of sex-matched ACM. **C** and ***D*,** Sex-matched ACM increased both frequency (***C***) and amplitude (***D***) of mEPSCs recorded from either male or female-derived cortical neurons. ***C’***, Fold-change of mEPSC frequency values (in Hz) induced by male or female-derived ACM (shown as percent of sex-matched NGM control); *p < 0.05, ***p < 0.001 (one-way ANOVA with Tukey’s multiple comparisons *post hoc* analysis; *F* = 10.29). ***D’***, Fold-change of mEPSC amplitude values (in pA) induced by male or female-derived ACM (shown as percent of sex-matched NGM control); *p < 0.05, **p < 0.01; n.s., not significant (Kruskal–Wallis test with Dunn’s multiple comparisons *post hoc* analysis; K-W statistic = 22.61).

Statistical analyses were performed in Microsoft Excel and GraphPad Prism and shown as mean ± SEM unless otherwise stated, with results summarized in Table 1. In order to calculate “% of NGM” values, co-localized puncta values (Fig. 1) or frequency/amplitude measurements (Fig. 2) for the same sex GM-only condition were averaged within an experimental replicate, then values across all other treatments/conditions were converted to a percentage of the calculated NGM average for that replicate. For estimation based on confidence intervals (CIs), we entered our raw data values into the appropriate module on https://www.estimationstats.com/ (Ho et al., 2019). Cumming estimation plots showing raw values, mean ± SD, and 95% CIs were downloaded directly from the website, along with the results of comparison testing. A total of 5000 bootstrap samples were taken; the CI is bias-corrected and accelerated. Effect sizes and CIs are reported as: effect size [CI width lower bound; upper bound]. The *p* value(s) reported are the likelihood(s) of observing the effect size(s), if the null hypothesis of zero difference is true. For each permutation *p* value, 5000 reshuffles of the control and test labels were performed.

**Table 1:** Statistical results for Western blottings, estradiol immunoassay, and ICC (fold-change and multiple group comparisons)

Figure	Figure panel	Statistical test	Conditions	Results
1	*B'*	Kruskal–Wallis test with Dunn’s *post hoc* comparison	M NGM vs M Astro	K-W = 65.23
				*p* < 0.0001
1	*B'*	Kruskal–Wallis test with Dunn’s *post hoc* comparison	F NGM vs F Astro	K-W = 65.23
				*p* < 0.0001
1	*B'*	Kruskal–Wallis test with Dunn’s *post hoc* comparison	M Astro vs F Astro	K-W = 65.23
				*p* > 0.9999
1	*D'*	Kruskal–Wallis test with Dunn’s *post hoc* comparison	M NGM vs M M-ACM	K-W = 77.03
				*p* < 0.0001
1	*D'*	Kruskal–Wallis test with Dunn’s *post hoc* comparison	M NGM vs M F-ACM	K-W = 77.03
				*p* < 0.0001
1	*D'*	Kruskal–Wallis test with Dunn’s *post hoc* comparison	M M-ACM vs M F-ACM	K-W = 77.03
				*p* > 0.9999
1	*D'*	Kruskal–Wallis test with Dunn’s *post hoc* comparison	M M-ACM vs F M-ACM	K-W = 77.03
				*p* = 0.0142
1	*D'*	Kruskal–Wallis test with Dunn’s *post hoc* comparison	M F-ACM vs F F-ACM	K-W = 77.03
				*p* = 0.1693
1	*D'*	Kruskal–Wallis test with Dunn’s *post hoc* comparison	F NGM vs F M-ACM	K-W = 77.03
				*p* = 0.2781
1	*D'*	Kruskal–Wallis test with Dunn’s *post hoc* comparison	F NGM vs F F-ACM	K-W = 77.03
				*p* = 0.0036
2	*C'*	One-way ANOVA with Tukey's *post hoc* comparison	M NGM vs M ACM	*F*_(3,75)_ = 10.29
				*p* = 0.0002
2	*C'*	One-way ANOVA with Tukey's *post hoc* comparison	F NGM vs F ACM	*F*_(3,75)_ = 10.29
	* *			*p* = 0.0195
2	*C'*	One-way ANOVA with Tukey's *post hoc* comparison	M ACM vs F ACM	*F*_(3,75)_ = 10.29
	* *			*p* = 0.2119
2	*D'*	Kruskal–Wallis test with Dunn’s *post hoc* comparison	M NGM vs M ACM	K-W = 22.61
	* *			*p* = 0.0165
2	*D'*	Kruskal–Wallis test with Dunn’s *post hoc* comparison	F NGM vs F ACM	K-W = 22.61
	* *			*p* = 0.0018
2	*D'*	Kruskal–Wallis test with Dunn’s *post hoc* comparison	M ACM vs F ACM	K-W = 22.61
	* *			*p* > 0.9999
4	*A*	Unpaired Student's *t* test	TSP2 relative band intensity, male vs female	*p* = 0.568
4	*B*	Two-way ANOVA (interaction)	α2δ−1 relative band intensity	*F*_(2,12)_ = 0.220
	* *			*p* = 0.805
4	*B*	Two-way ANOVA (sex)	α2δ−1 relative band intensity	*F*_(1,12)_ = 0.647
	* *			*p* = 0.437
4	*B*	Two-way ANOVA (treatment)	α2δ−1 relative band intensity	*F*_(2,12)_ = 0.093
	* *			*p* = 0.912
4	*C*	Linear mixed-effects model (REML) with Holm–Sidak’s *post hoc* comparison (sex)	E2 (pg/ml)	*F*_(1,55)_ = 6.999
	* *			*p* = 0.011
4	*D*	Linear mixed-effects model (REML) with Holm–Sidak’s *post hoc* comparison (sex)	E2 (pg/ml)	*F*_(1,29)_ = 3.799
				p = ns
5	*E*, left	Kruskal–Wallis test with Dunn’s *post hoc* comparison	NGM vs Astro	K-W = 52.57
				*p* = 0.0002
5	*E*, left	Kruskal–Wallis test with Dunn’s *post hoc* comparison	NGM vs Astro + Let	K-W = 52.57
				*p* > 0.9999
5	*E*, left	Kruskal–Wallis test with Dunn’s *post hoc* comparison	NGM vs ACM	K-W = 52.57
				*p* = 0.0002
5	*E*, left	Kruskal–Wallis test with Dunn’s *post hoc* comparison	NGM vs ACM + Let	K-W = 52.57
				*p* > 0.9999
5	*E*, left	Kruskal–Wallis test with Dunn’s *post hoc* comparison	Astro vs Astro + Let	K-W = 52.57
				*p* = 0.0198
5	*E*, left	Kruskal–Wallis test with Dunn’s *post hoc* comparison	ACM vs ACM + Let	K-W = 52.57
				*p* < 0.0001
5	*E*, right	Kruskal–Wallis test with Dunn’s *post hoc* comparison	NGM vs Astro	K-W = 66.18
				*p* < 0.0001
5	*E*, right	Kruskal–Wallis test with Dunn’s *post hoc* comparison	NGM vs Astro + Let	K-W = 66.18
				*p* = 0.0123
5	*E*, right	Kruskal–Wallis test with Dunn’s *post hoc* comparison	NGM vs ACM	K-W = 66.18
				*p* < 0.0001
5	*E*, right	Kruskal–Wallis test with Dunn’s *post hoc* comparison	NGM vs ACM + Let	K-W = 66.18
				*p* > 0.9999
5	*E*, right	Kruskal-Wallis test with Dunn’s *post hoc* comparison	Astro vs Astro + Let	K-W = 66.18
				*p* = 0.2655
5	*E*, right	Kruskal-Wallis test with Dunn’s *post hoc* comparison	ACM vs ACM + Let	K-W = 66.18
				*p* < 0.0001
6	left	Kruskal–Wallis test with Dunn’s *post hoc* comparison	NGM vs TSP2	K-W = 64.21
				*p* = 0.0039
6	left	Kruskal–Wallis test with Dunn’s *post hoc* comparison	NGM vs E2	K-W = 64.21
				*p* = 0.2220
6	left	Kruskal–Wallis test with Dunn’s *post hoc* comparison	NGM vs E2 TSP2	K-W = 64.21
				*p* > 0.9999
6	left	Kruskal–Wallis test with Dunn’s *post hoc* comparison	NGM vs ICI	K-W = 64.21
				*p* = 0.0452
6	left	Kruskal–Wallis test with Dunn’s *post hoc* comparison	NGM vs ICI TSP2	K-W = 64.21
				*p* = 0.2420
6	left	Kruskal–Wallis test with Dunn’s *post hoc* comparison	NGM vs PGE2	K-W = 64.21
				*p* = 0.0377
6	left	Kruskal–Wallis test with Dunn’s *post hoc* comparison	NGM vs PGE2 TSP2	K-W = 64.21
				*p* = 0.0467
6	left	Kruskal–Wallis test with Dunn’s *post hoc* comparison	E2 vs E2 TSP2	K-W = 64.21
				*p* > 0.9999
6	left	Kruskal–Wallis test with Dunn’s *post hoc* comparison	ICI vs ICI TSP2	K-W = 64.21
				*p* > 0.9999
6	left	Kruskal–Wallis test with Dunn’s *post hoc* comparison	PGE2 vs PGE2 TSP2	K-W = 64.21
				*p* > 0.9999
6	right	Kruskal–Wallis test with Dunn’s *post hoc* comparison	NGM vs TSP2	K-W = 44.75
				*p* > 0.9999
6	right	Kruskal–Wallis test with Dunn’s *post hoc* comparison	NGM vs E2	K-W = 44.75
				*p* > 0.9999
6	right	Kruskal–Wallis test with Dunn’s *post hoc* comparison	NGM vs E2 TSP2	K-W = 44.75
				*p* > 0.9999
6	right	Kruskal–Wallis test with Dunn’s *post hoc* comparison	NGM vs ICI	K-W = 44.75
				*p* = 0.8729
6	right	Kruskal–Wallis test with Dunn’s *post hoc* comparison	NGM vs ICI TSP2	K-W = 44.75
				*p* > 0.9999
6	right	Kruskal–Wallis test with Dunn’s *post hoc* comparison	NGM vs PGE2	K-W = 44.75
				*p* > 0.9999
6	right	Kruskal–Wallis test with Dunn’s *post hoc* comparison	NGM vs PGE2 TSP2	K-W = 44.75
				*p* = 0.0002
6	right	Kruskal–Wallis test with Dunn’s *post hoc* comparison	E2 vs E2 TSP2	K-W = 44.75
				*p* > 0.9999
6	right	Kruskal–Wallis test with Dunn’s *post hoc* comparison	ICI vs ICI TSP2	K-W = 44.75
				*p* = 0.6315
6	right	Kruskal–Wallis test with Dunn’s *post hoc* comparison	PGE2 vs PGE2 TSP2	K-W = 44.75
				*p* < 0.0001

## Results

### Astrocytic contributions to cortical synaptogenesis are differentially regulated between sexes

To elucidate sex differences in astrocyte-mediated synaptogenesis, we isolated cortical neurons and astrocytes with >95% purity from P1 Sprague Dawley rat male or female pups. Neurons were cultured for two weeks in complete NGMs, treated with either astrocyte culture inserts (Astro) or ACM. Following immunocytochemical (ICC) staining, presynaptic (Bassoon) and postsynaptic (Homer1) puncta were imaged and their co-localization quantified to determine excitatory synapse number in the various treatment conditions (Fig. 1*A*). Astrocyte inserts allow for continuous exchange of secreted factors between astrocytes and neurons via shared media. Both male and female-derived cortical neurons showed a significant increase in excitatory synapse density along their processes when cultured with sex-matched astrocyte inserts (Fig. 1*A*,*B*). The unpaired mean difference between M NGM and M Astro was 31.0 [95.0%CI 17.4, 45.8]; between F NGM and F Astro, the difference was 23.2 [95.0%CI 9.28, 40.3]. The *p* values of the two-sided permutation *t* tests were 0.0 and 0.0028, respectively. The percent change (compared with NGM only) of the astrocyte-induced synapses in female cultures was not as high as that in male cultures (221 ± 20% compared with 279 ± 28%, respectively), though this difference was not significant (Fig. 1*B’*). To determine whether secreted factors from astrocytes differed in their synaptogenic potential depending on the sex of the donors, we treated male and female-derived cortical neurons with ACM from either male (M-ACM) or female (F-ACM) astrocytes. Excitatory synapse density was increased in neurons cultured from both sexes, with male and female cultures responding significantly to sex-matched ACM (Fig. 1*C*,*D*). In the male cultures, the unpaired mean difference between NGM and M-ACM was 18.1 [95.0%CI 12.3, 24.4], while between NGM and F-ACM it was 23.5 [95.0%CI 15.2, 33.3]; the *p* values of the two-sided permutation *t* tests were both 0.0. In the female cultures, these same comparisons resulted in differences of 6.09 [95.0%CI −2.7, 14.3] and 14.7 [95.0%CI 4.73, 25.1], respectively (permutation *t* test values were 0.163 and 0.0046, respectively). Interestingly, however, the percent change of the increase in synapses was significantly higher in male compared with female neurons for M-ACM (207 ± 14% in male cultures compared with 157 ± 19% in female cultures; *p* < 0.05) and less so for F-ACM (218 ± 19% male, 162 ± 14% female; *p* = 0.169, Kruskal–Wallis test with Dunn’s multiple comparisons *post hoc* analysis; K-W statistic = 77.03 (Fig. 1*D’*). These results indicate that cortical neurons respond to astrocyte-secreted synaptogenic factors at different rates depending on sex.

We next wanted to test whether astrocyte-induced synaptic activity was differently impacted by sex. Male and female cortical neurons were patched in whole-cell configuration and mEPSCs were recorded to assess baseline synaptic activity (Fig. 2*A*,*B*). Both mEPSC frequency (Fig. 2*C*) and amplitude (Fig. 2*D*) increased in response to sex-matched ACM treatment in males and females. Unpaired mean differences between NGM and ACM for frequency comparisons were 0.774 [95.0%CI 0.432, 1.24] for males and 0.783 [95.0%CI 0.325, 1.36] for females, with two-sided permutation *t* test *p* values were 0.0008 and 0.0038, respectively. For amplitude, unpaired mean differences were 5.94 [95.0%CI 2.84, 9.45] for males and 8.98 [95.0%CI 3.28, 14.9] for females, with *p* values of 0.0022 and 0.0066, respectively. Comparison of fold-changes indicates a larger magnitude ACM-induced increase in frequency in male neurons compared with female (2.74 ± 0.46 compared with 2.01 ± 0.25, respectively), though this did not result in a significant difference (Fig. 2*C’*). The fold-changes in amplitude increase were similar (1.61 ± 0.15 male, 1.46 ± 0.12 female; Fig. 2*D’*), suggesting that potential sex differences in astrocyte-mediated excitatory synaptogenesis manifest primarily as changes in synapse number rather than strength.

### TSP promotes synaptic development in male but not female cortical neurons

**Figure 3.**
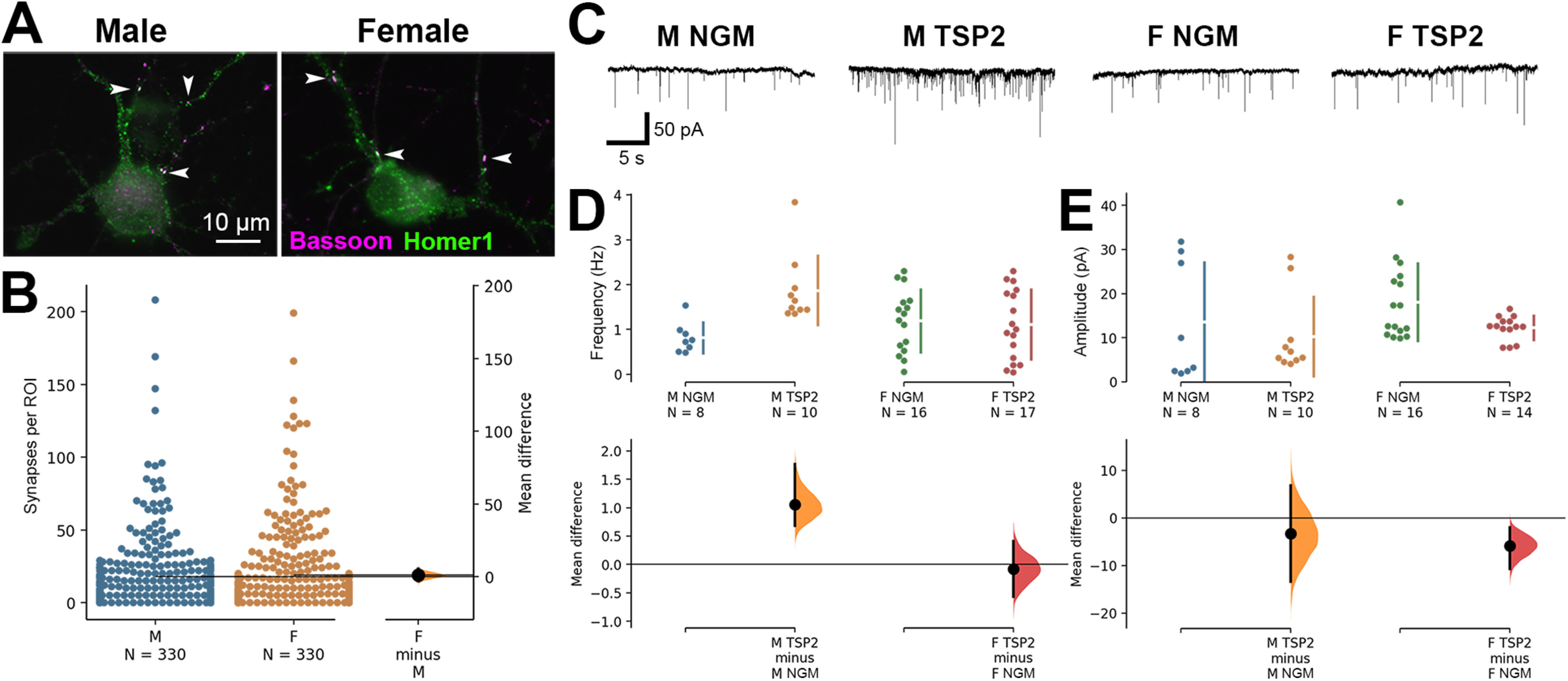
Increased synaptic response to astrocytic factor TSP2 by male but not female-derived cortical neurons. ***A***, Representative ICC images of male and female-derived cortical neurons. Co-localized presynaptic (Bassoon; magenta) and postsynaptic (Homer1; green) puncta reveal sites of excitatory synapses (white arrowheads). ***B***, No baseline difference in the number of co-localized excitatory synaptic puncta between male and female-derived neurons with standard NGM treatment. The mean difference between M and F is shown in the Gardner–Altman estimation plot. Both groups are plotted on the left axes; the mean difference is plotted on a floating axis on the right as a bootstrap sampling distribution. The mean difference is depicted as a dot; the 95% CI is indicated by the ends of the vertical error bar. ***C***, Sample mEPSC traces from male and female-derived cortical neurons treated with either standard NGM or NGM with the addition of purified TSP2. ***D***, ***E***, TSP2 treatment increased frequency (***D***) but not amplitude (***E***) of mEPSCs recorded from male-derived cortical neurons. Female-derived cortical neurons showed no change in mEPSC frequency but had a small decrease in amplitude after TSP2.

To rule out the possibility that inherent sex differences in synapse density are responsible for the distinct effects of astrocyte-secreted factors on synaptic development, we compared basal synapse number between male and female-derived cortical neuron cultures via ICC (Fig. 3*A*,*B*). No differences were found in synaptic density between the sexes (0.578 [95.0%CI −2.09, 3.35]; *p* = 0.684), indicating that the differences we previously observed must have been because of a differential response to astrocytic factors.

TSPs were among the first astrocyte-secreted factors identified as having synaptogenic properties (Christopherson et al., 2005; Risher and Eroglu, 2012). However, this and other previous studies investigating the synapse-promoting properties of TSP were performed using neurons isolated from mixed-sex litters. To specifically test whether there were sex differences in TSP-induced synaptogenesis, we next analyzed mEPSCs from male versus female cultures treated with purified TSP2 (Fig. 3*C*), one of the five synaptogenic TSP isoforms expressed in mammals. Intriguingly, only male-derived neurons showed increased mEPSC frequency after TSP2 treatment (Fig. 3*D*). The unpaired mean difference between M GM and M TSP2 was 1.06 [95.0%CI 0.68, 1.77]; for F GM and F TSP2: −0.0838 [95.0%CI −0.569, 0.409]; *p* values for the two-sided permutation *t* tests were 0.001 and 0.742, respectively. Male neurons showed no change in mEPSC amplitude following TSP2 treatment, while female neurons actually showed a slight decrease (M: −3.27 [95.0%CI −13.4, 6.82] with *p* = 0.546; *F* = −5.82 [95.0%CI −10.7, −2.02] with *p* = 0.017; Fig. 3*E*), this finding was consistent with previous studies showing that TSPs promote the formation of NMDA-containing silent synapses that lack functional AMPA receptors (Risher and Eroglu, 2012). Nevertheless, these results indicate that TSP2 is synaptogenic in male but not female cortical neurons, providing a potential mechanism underlying the differential rates of astrocyte-induced synaptic development between the sexes.

### Modulation of TSP-induced synaptogenesis by estrogen

**Figure 4.**
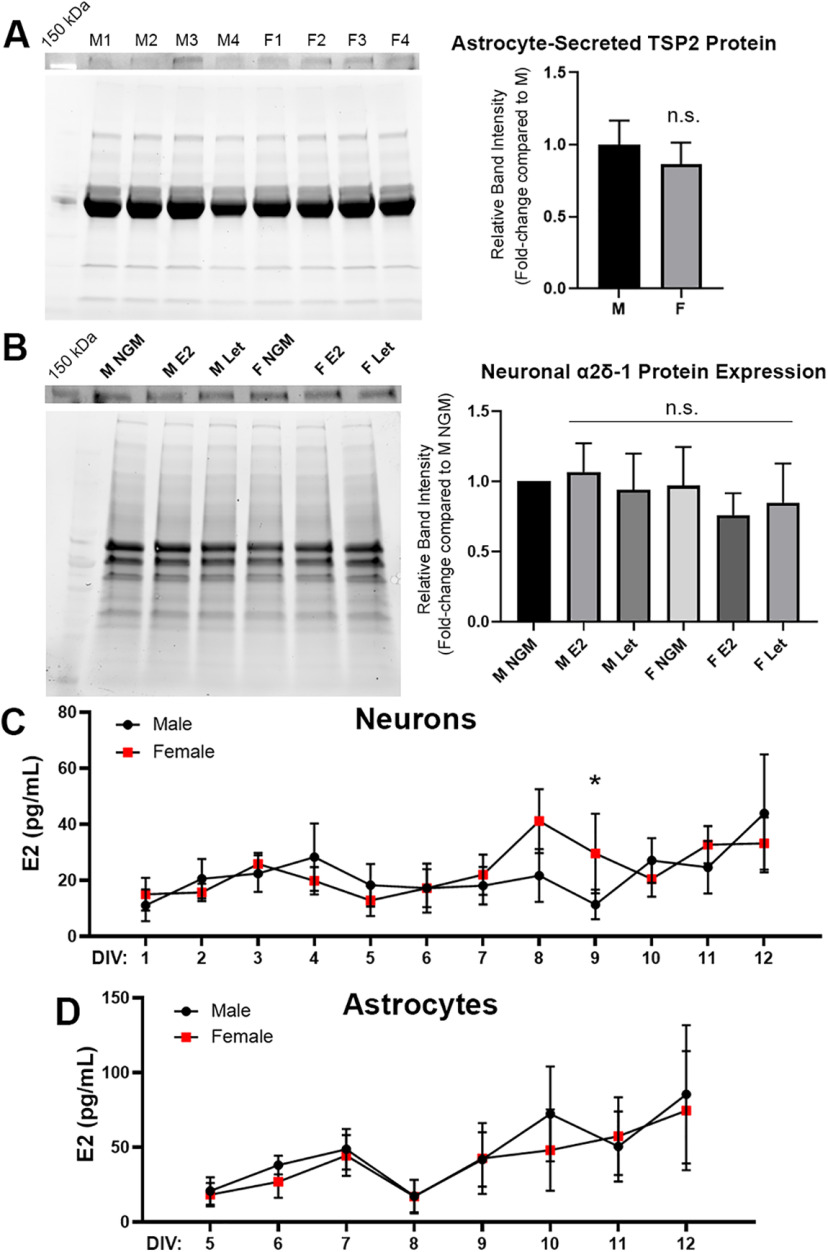
Transient increase in neuronally-derived estradiol in cultured female cortical neurons. ***A***, left, Western blotting for TSP2 [top, ∼160-kDa observed band (∼130 kDa expected); bottom, total protein blot for normalization] from astrocyte media collected on DIV10. Right, No difference was found in secreted TSP2 levels between male and female astrocytes; *p* = 0.568 [unpaired Student’s *t* test; *n* = 4 independent experimental replicates (M1-4, F1-4)]. ***B***, left, Western blotting for α2δ−1 (top, 143-kDa observed band; bottom, total protein blot for normalization) from purified male and female cortical neuron lysates. Lysates were collected on DIV13 following treatment with NGM only, 100 nm E2, or 100 nm letrozole (Let) on DIV7 and DIV10. Right, No difference in total α2δ−1 protein expression was observed between male or female-derived neurons at baseline or following treatment with either E2 or Let; *p* = 0.805 (two-way ANOVA, interaction: *F*_(2,12)_ = 0.220; *n* = 3 independent experimental replicates). ***C***, Immunoassay results for levels of E2 detected in male versus female neuron-conditioned media. An increase in secreted E2 was detected in female cultures at the start of the second week, reaching significance at DIV9; *p < 0.05 [linear mixed-effects model (REML) with Holm–Sidak’s *post hoc* analysis, sex: *F*_(1,55)_ = 7.00; *n* = 4 independent experimental replicates]. ***D***, Estradiol measurements from male versus female cortical ACM. No significant differences were observed (linear mixed-effects model, sex: *F*_(1,29)_ = 3.80; *n* = 3 independent experimental replicates).

Why is TSP effective in promoting synapse formation in male but not female neurons? To begin to address this question, we performed Western blotting to rule out that there were significant sex differences with either astrocytic secretion of TSP2 or expression of the synaptogenic TSP2 receptor, α2δ−1 (Eroglu et al., 2009). Media collected from cultured astrocytes showed no difference in secreted TSP2 levels between sexes (Fig. 4*A*), while α2δ−1 was similarly expressed in both male and female-derived neuron cultures (Fig. 4*B*). We then asked whether the unique responses of these cultures were because of differences in the sex hormone estrogen since, like astrocytes, sex hormones have been found to be powerful regulators of synaptic connectivity. Fluctuating levels of circulating estrogen, and androgens to a lesser extent, can drive significant changes in synaptic density in both sexes (Woolley and McEwen, 1992; Leranth et al., 2003; Srivastava and Penzes, 2011). Neurons, including those in dissociated culture, are capable of *de novo* production and secretion of estrogen (Prange-Kiel et al., 2003; Hojo et al., 2004), which is enzymatically converted from the hormone testosterone by aromatase. We therefore investigated whether application of 17β-estradiol (E2), the predominant biologically active form of estrogen, or letrozole, an aromatase inhibitor, would affect neuronal α2δ−1 protein expression. However, neither E2 nor letrozole had any effect on α2δ−1 protein levels in our cultures (Fig. 4*B*). We then asked whether there were more subtle differences in endogenous E2 levels that may help explain the TSP2 sex discrepancy. Toward this possibility, Konkle and McCarthy (2011) previously showed that cortical levels of E2 are transiently higher in female rats than males by the end of the first postnatal week. We observed a similar effect in our cortical neuron cultures, reaching significance at DIV9 (Fig. 4*C*). Interestingly, this time point corresponded with the peak period of synaptogenesis induced by TSPs, as well as the normal developmental expression of TSP1 and TSP2 (Christopherson et al., 2005; Risher and Eroglu, 2012) and α2δ−1 (Risher et al., 2018). By contrast, astrocytes, which have also been identified as sources of estrogen in the brain (Azcoitia et al., 2011), did not show any sex differences in E2 secretion over this same time period (Fig. 4*D*). These findings suggested that neuronally-derived E2 may be a critical regulator of TSP-induced synaptogenesis.

**Figure 5.**
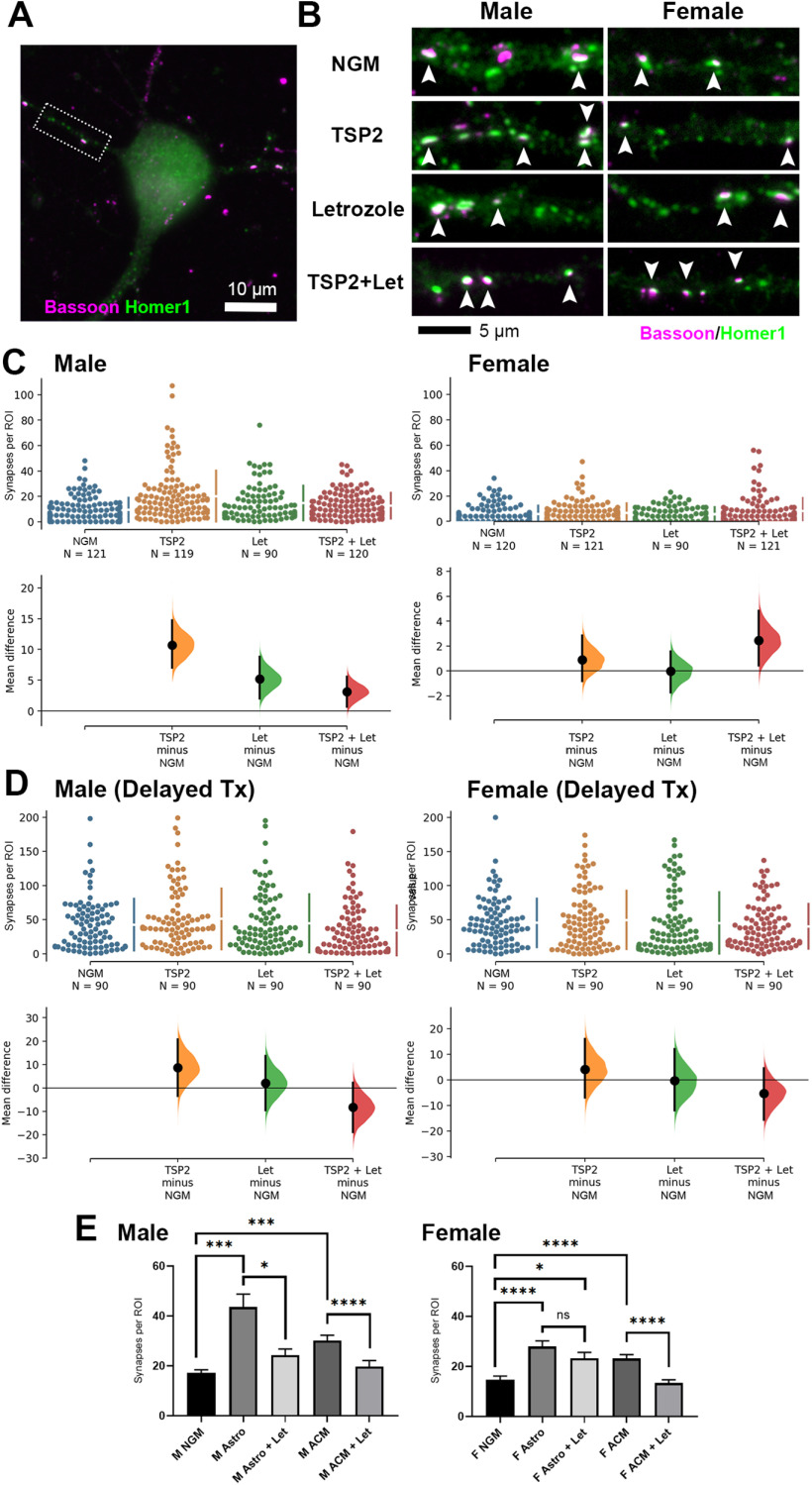
Inhibition of neuronal estrogen production modulates TSP/astrocyte-induced synaptogenesis. ***A***, ICC image of a male-derived cortical neuron fixed and stained on DIV13 with presynaptic (Bassoon; magenta) and postsynaptic (Homer1; green) markers. Dotted box indicates example dendritic ROI sampled for images in ***B***, which shows co-localized excitatory synaptic puncta (white, arrowheads) along male and female-derived cortical neurites treated with NGM only or NGM plus TSP2, 100 nm letrozole (Let), or TSP2/letrozole on DIV7 and DIV10. ***C***, left, Letrozole had a mild synaptogenic effect on male-derived cortical neurons but attenuated the synapse-promoting ability of TSP2. Right, Female-derived cortical neurons showed no synaptogenic response to TSP2 unless treatment was combined with Let. ***D***, Delayed treatment (DIV13 and DIV16, fixed and stained DIV19) revealed no differences in synapse number from NGM-only condition following TSP2 and/or Let treatment in neurons from either sex. ***E***, Quantification of Bassoon/Homer1 co-localized synaptic puncta following DIV7/DIV10 treatment schedule showed that Let abolished the synaptogenic effects of sex-matched astrocyte inserts (Astro) and ACM in male-derived neurons as well as ACM in female-derived neurons. No attenuation was observed with Let and Astro in females; *p < 0.05, ***p < 0.001, ****p < 0.0001 [Kruskal–Wallis test with Dunn’s multiple comparisons *post hoc* analysis; K-W statistic = 52.57 (male), 66.18 (female); *n* = 30 cells per condition per experimental replicate, 3 independent experimental replicates].

To investigate whether neuronally-synthesized estrogen interferes with TSP-induced synapse formation, we used ICC to quantify excitatory synapses in our purified cortical neuron cultures (Fig. 5*A*,*B*) following treatment with TSP2 and letrozole. Following nearly two weeks of *in vitro* culture, ICC strengthened our electrophysiological findings (Fig. 3*D*) by showing that TSP2 significantly increased synapse numbers in male but not female-derived neurons (Fig. 5*C*). Intriguingly, treatment with letrozole revealed a sex-divergent effect of estrogen inhibition on the synapse-promoting ability of TSP2. Letrozole facilitated TSP2-induced synaptogenesis in female-derived neurons, albeit not to the same degree as in neurons isolated from males (Fig. 5*C*). The most unexpected result was seen in the male cultures, where letrozole seemed to have the opposite effect, greatly decreasing the magnitude of synaptogenesis induced by TSP2 although it was mildly synaptogenic on its own (Fig. 5*C*). However, the combined effect of TSP2 and letrozole still led to increased synapses over the control treatment in male-derived cells, but now the percent change of this increase was virtually indistinguishable from that seen in the female cultures (164 ± 15% male, 160 ± 21% female). In male cultures, the unpaired mean difference between NGM and TSP2 was 10.7 [95.0%CI 7.03, 14.7], between NGM and Let was 5.18 [95.0%CI 2.06, 8.83], and between NGM and TSP2+Let was 3.13 [95.0%CI 0.685, 5.56] (*p* values for the two-sided permutation *t* tests were 0.0, 0.0012, and 0.0116, respectively). In female cultures, the mean differences for those same comparisons were 0.903 [95.0%CI −0.808, 2.85], −0.0306 [95.0%CI −1.72, 1.57], and 2.46 [95.0%CI 0.436, 4.84] (*p* values of 0.338, 0.962, and 0.0292, respectively). To test whether the sex difference in the TSP2 response was limited to this specific time period when E2 was transiently elevated in female cultures (Fig. 4*C*), we performed ICC on neurons with delayed TSP2 and/or letrozole treatment. Compared with our standard treatment timeline (DIV7–DIV13), this delayed treatment timeline was performed between DIV13 and DIV19, well after the sex difference in E2 levels had dissipated. We observed no differences from baseline with TSP2 or letrozole in either sex with the delayed treatment (Fig. 5*D*), supporting the existence of a sex-specific critical window for TSP2-induced synapse formation during the second postnatal week that coincides with the transient difference in E2 levels. For male cultures that received the delayed treatment, the unpaired mean difference between NGM and TSP2 was 8.58 [95.0%CI −3.36, 20.7], between NGM and Let was 1.99 [95.0%CI −9.49, 13.7], and between NGM and TSP2+Let was −8.31 [95.0%CI −18.9, 2.28] (*p* values for the two-sided permutation *t* tests were 0.167, 0.755, and 0.138, respectively). In female cultures with delayed treatment, the mean differences for those same comparisons were 4.1 [95.0%CI −6.94, 16.0], −0.389 [95.0%CI −11.9, 12.0], and −5.27 [95.0%CI −15.6, 4.49] (*p* values of 0.485, 0.947, and 0.299, respectively).

Taken together, these results demonstrate clear sex differences in TSP2-induced synaptogenesis that may be regulated, at least in part, by estrogen synthesized endogenously by neurons. To determine whether decreasing estrogen synthesis would specifically interfere with TSP2 signaling pathways or with astrocyte-mediated synaptogenesis in general, we applied letrozole to male and female-derived neuron cultures that were being treated with sex-matched astrocyte inserts or ACM (Fig. 5*E*). In these treatments, we indeed observed a strong overall attenuation of astrocyte/ACM-induced synapses with letrozole in a similar manner to the reduction seen with TSP2 plus letrozole. Interestingly, letrozole treatment did not reduce the increase in synapses promoted by astrocytes in the female-derived cultures, raising the possibility that inhibiting E2 synthesis may have affected astrocytes in a sex-specific fashion.

**Figure 6.**
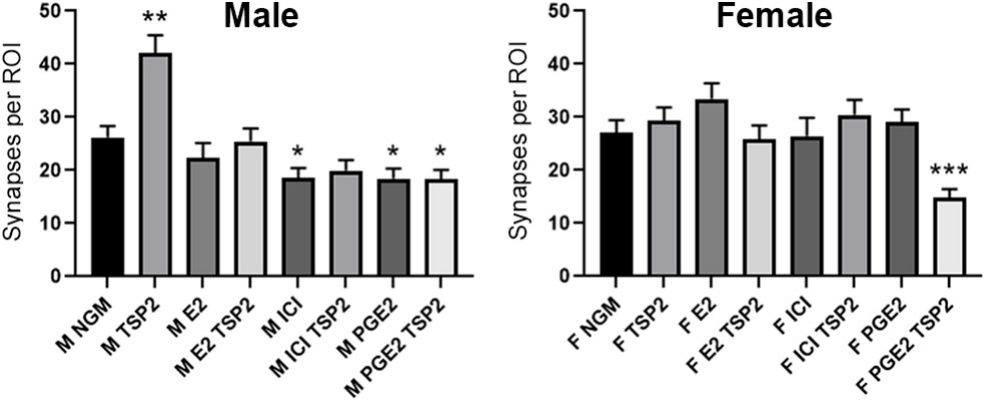
Endogenous estrogen signaling permits TSP2-induced synapse formation in male but not female neurons. Quantification of Bassoon/Homer1 co-localized synaptic puncta following DIV7/DIV10 treatment schedule showed that modulating endogenous estrogen signaling, either with exogenous 100 nm E2, 100 nm ICI 182780 (ER antagonist), or 100 nm PGE2 (aromatase agonist), precluded the ability of TSP2 to promote synapse formation in either male (left)-derived or female (right)-derived cortical neurons. PGE2, in particular, was notable in that it lowered synapse number compared with NGM alone in males and when combined with TSP2 in both sexes; *p < 0.05, **p < 0.01, ***p < 0.001 [Kruskal–Wallis test with Dunn’s multiple comparisons *post hoc* analysis; K-W statistic = 64.21 (male), 44.75 (female); *n* = 30 cells per condition per experimental replicate, 3 independent experimental replicates].

We next tested the extent to which TSP2-induced synaptogenesis could be affected by modulating neuronal estrogen signaling beyond the drug letrozole. Using the male and female-derived cortical neuron cultures, we combined TSP2 treatment with one of three other factors involved with estrogen signaling: exogenous E2, ICI 182780 (an estrogen receptor α/β antagonist), and prostaglandin E2 (PGE2; aromatase agonist). Whereas inhibiting aromatase with letrozole mildly increased synapse number when combined with TSP2 in both male and female neurons (Fig. 5*C*), stimulating aromatase activity with PGE2 resulted in a net decrease in synapses with TSP2 treatment (Fig. 6). Furthermore, strongly manipulating estrogen signaling in either direction, either stimulating by adding exogenous E2 or inhibiting estrogen receptors with ICI 182780, completely precluded the ability of TSP2 to promote synaptogenesis in either sex (Fig. 6). Combined, these results show that estrogen and TSP2/astrocyte signaling are tightly interlinked in the differential regulation of synaptic connectivity between males and females.

## Discussion

Female and male brains show fundamental differences throughout development and evolution. The adult human female brain estimated to contain approximately two-thirds the number of synapses across all cortical layers compared with males (Alonso-Nanclares et al., 2008), raising the possibility of fundamentally sex-distinct mechanisms of how synaptic networks are formed, developed, and maintained. Here, we provide evidence for one such mechanism, whereby cortical neurons purified from males, but not females, are highly receptive to the astrocyte-secreted synaptogenic factor TSP2. Disruption of neuronal estradiol synthesis abolished this sex difference and resulted in TSP2 being weakly synaptogenic for both sexes. In addition, significantly modulating estrogen signaling, positively or negatively (via exogenous E2 or blocking ERs, respectively), completely prevents TSP2-induced synaptogenesis, highlighting endogenous brain estrogen as a potentially critical player in astrocyte/synaptic signaling.

TSP is a potent synaptic organizer secreted by astrocytes to act through the neuronal L-type calcium channel auxiliary subunit, α2δ−1, during a peak period of synaptogenesis (Eroglu et al., 2009; Risher and Eroglu, 2012). This period consists of roughly the second to third postnatal weeks of rodent brain development, coinciding with a stage of rapid astrocyte growth and elaboration (Farhy-Tselnicker and Allen, 2018) that is thought to be roughly correlated with perinatal human brain development (Semple et al., 2013). Since this time point falls well before the onset of puberty (typically between days 34 and 48 in rats), neurosteroids have not traditionally been considered to play a significant role in shaping synaptic connectivity. Though we cannot rule out potential contributions from the gonadal testosterone surge near the end of gestation in male rats (Weisz and Ward, 1980), our observation of significant sex differences before the developmental milestone of gonadal hormone release, and the elimination of this difference by letrozole, suggests that our findings are likely because of *de novo* estrogen synthesis occurring in the very young brain. The phenomenon of *de novo* estrogen production by brain cells has previously been reported (McCarthy, 2008), but the importance of this process for proper neural circuit formation and operation had not been determined.

Our results so far certainly indicate that neuronally-sourced estradiol regulates astrocyte-mediated synaptic development, though defining the precise nature of this role has been complicated by the seemingly contradictory finding that blocking aromatase reduces the synaptic capability of TSP2 in male-derived neurons yet is permissive in female cultures. Furthermore, stimulating aromatase via PGE2 appeared to have a negative effect on synaptogenesis in male neurons, while combining PGE2 with TSP2 resulted in decreased synapse number in both sexes (Fig. 6). A number of groups have previously investigated sex differences in aromatase expression throughout the CNS (Bender et al., 2017; Fels et al., 2021; Tabatadze et al., 2014). Though the studies varied in terms of age as well as methodology (mRNA vs protein), a common conclusion is that sex differences do exist in some brain regions but not others. Cortex and hippocampus, for example, are typically cited as areas that do not show significant sex differences in aromatase expression, yet other areas such as the preoptic area of the hypothalamus and the bed nucleus of the stria terminalis (BNST) present with strong sex biases. Since the list of CNS functions influenced by estrogen signaling is quite extensive (McCarthy, 2008), it would stand to reason that blocking aromatase would have widespread consequences that go far beyond the synaptogenic mechanism that we are investigating here. However, there are precedents for sex-specific synaptic responses to letrozole in the hippocampus and basolateral amygdala, despite similar levels of aromatase expression (Bender et al., 2017; Vierk et al., 2012). Further complicating the story is the knowledge that estradiol would not be the sole compound whose synthesis would be affected by letrozole, since aromatase inhibition would make testosterone, the substrate for E2, more readily available for conversion to dihydrotestosterone (DHT) via 5α-reductase. DHT acts via androgen receptors (ARs), which have previously been shown to influence synaptic development in a sex-specific manner (Brandt et al., 2020). Studies are ongoing to elucidate this mechanism more fully, as well as to establish the relevance of this process for sex differences in cortical development *in vivo*.

The role of estrogen as a master regulator of synaptic connectivity has been well-established (for review, see Grkovic and Mitrovic, 2020). However, many previous studies in the field that established the effects of estrogen on synapse formation were performed in conditions that included glia, such as intact brain, hippocampal slice culture, or dissociated neuronal culture with up to 20% glial contamination (Kretz et al., 2004; Mukai et al., 2007; Kramár et al., 2009; Zhao et al., 2018; Lu et al., 2019; Brandt and Rune, 2020). Both neurons and astrocytes express estrogen receptors (Hösli and Hösli, 1999; Hösli et al., 2001), while astrocytes in particular can rapidly respond to fluctuating levels of estrogen (Chaban et al., 2004), suggesting a potential confound for these findings. In our neuronal cultures, we achieve 95% purity (confirmed by ICC) and include cytosine arabinoside (AraC) to inhibit the proliferation of the astrocytes that are present. In this way, we are able to isolate the responses of neurons and specifically provide the factors typically sourced from astrocytes, either as individual components (i.e., TSPs) or as a heterogeneous mixture (i.e., ACM). As the findings in this study have indicated, such an approach may be necessary to continue unraveling the intersection between estrogen and astrocytes in the shaping of synaptic connectivity (McCarthy et al., 2003).

In addition to the known differences between male and female brains of healthy individuals, aberrant brain states are also known to manifest differently between the sexes. Diseases of the aging brain, such as Alzheimer’s and Parkinson’s, present with highly different rates of incidence and severity between men and women, while many more boys than girls are diagnosed with certain neurodevelopmental disorders (NDDs) including schizophrenia and autism spectrum disorder (Abel et al., 2010; Werling and Geschwind, 2013; Hanamsagar and Bilbo, 2016). In the case of NDDs, it is tempting to speculate that early dysregulation of synaptic connectivity may contribute to disease pathogenesis in a sex-dependent manner. Indeed, synaptic pathology and aberrant spinogenesis are common findings in both autism and schizophrenia, with a number of overlapping candidate molecules between the disorders (including TSP receptor α2δ−1 and its associated calcium channel subunits) that suggest some degree of common mechanistic dysfunction (Penzes et al., 2011; Iossifov et al., 2014; Risher et al., 2018; Harrison et al., 2020). Future studies that aim to elucidate disease pathology for the sake of developing novel, targeted therapies (Gillies and McArthur, 2010) should therefore take into consideration whether astrocyte-mediated synaptic signaling pathways are differentially regulated and/or disturbed in one sex compared with the other.

In summary, our findings have revealed that at least one prominent synaptogenic pathway regulated by astrocytes displays a significant sex bias, with TSP2 promoting synapse formation in male but not female cortical neurons. Future studies investigating other astrocyte factors, such as TSP1 and TSP4, hevin (Risher et al., 2014), SPARC (Kucukdereli et al., 2011), glypicans (Allen et al., 2012; Farhy-Tselnicker et al., 2017), and TGF-β (Diniz et al., 2014), among others, may determine whether this phenomenon affects other glial signaling pathways or is specific to TSP2. From this starting point, we can start to more thoroughly investigate how astrocytes promote synaptic connectivity in both sexes, elucidating potential mechanisms that underlie fundamental differences in the male and female brain to address an understudied yet vitally important area of neuroscience (McCarthy et al., 2012).

## Synthesis

Reviewing Editor: Nathalie Rouach, College of France Decisions are customarily a result of the Reviewing Editor and the peer reviewers coming together and discussing their recommendations until a consensus is reached. When revisions are invited, a fact-based synthesis statement explaining their decision and outlining what is needed to prepare a revision will be listed below. The following reviewer(s) agreed to reveal their identity: NONE.

Reviewer 1 :

In this study, the authors reveal a surprising sex difference in the regulation of synaptic connectivity by astrocyte-derived secreted factors. The authors find that synaptogenesis in female cortical neurons is less responsive to secreted factors from cocultured astrocytes or from astrocyte-conditioned medium. Strikingly, treatment of male and female cortical neuron cultures with the astrocyte-derived secreted factor Thrombospondin-2 (TSP2) reveals that only male neurons increase their synapse density in response to TSP2. The authors then explore a potential contribution of sex hormones to these differences. They identify a small transient increase in estradiol levels in female neurons at DIV9, whereas glial levels are similar between male and female. Finally, the authors use letrozole, an inhibitor of the enzyme that converts testosterone to estrogen. This treatment slightly increases TSP2’s synaptogenic effect in female neurons. Surprisingly and unexpectedly, this treatment blocks TSP2-induced synaptogenesis in male neurons. Overall, this is an interesting study that emphasizes the need to take sex differences into account for studies investigating synaptogenic factors. The exact role of estrogen in TSP2-mediated synaptogenesis remains unclear.

Specific comments:

The role of estrogen is unclear. I don’t expect the authors to fully elucidate the underlying mechanism, but additional insight is necessary.

1. There is a small difference in male vs female estradiol levels in neurons at DIV9 and blocking estrogen synthesis mildly promotes TSP2-mediated synaptogenesis in female neurons. Would TSP2 treatment of cortical neurons at an earlier stage in vitro (e.g. DIV2-7), when estradiol levels are equally low in male and female neurons, have a synaptogenic effect on female neurons?

2. Are there differences in male vs female expression of the alpha2delta-1 TSP2 receptor (or additional TSP receptors)? Potential differences in expression (mRNA and protein) might help explain sex-specific effects of TSP2.

3. Does estrogen affect alpha2delta-1 receptor levels? Does aromatase block affect alpha2delta-1 expression, especially in male neurons?

4. Some additional discussion on potential effects of aromatase blocking in male neurons would be helpful. Is aromatase equally expressed in male and female neurons? Would this block affect other processes in the cell?

5. It would be helpful to indicate how long cultures were treated with TSP2 in the legends of Figures 2 and 4. From the Methods it seems treatment was done on DIV7 and DIV10, and analysis on DIV13, but please specify for ease of viewing.

Reviewer 2 :

In this manuscript the authors examine the sex differences in synapse regulation by astrocytes. The authors show that media collected from cultured astrocytes from either sex, induces formation of more synapses in males than in females. The authors then examine the effects of an astrocyte secreted synapse regulating factor Thrombospondin 2 (TSP2), and find that it selectively promotes synapse formation in males, providing possible explanation for the differences seen in neurons treated with the astrocyte culture media. To identify the underlying mechanism the authors analyzed changes in levels of the sex hormone estrogen, and observed transiently higher levels of estrogen in female neurons. Treatment of female neurons with estrogen synthesis blocker letrozole, increased their response to synapse promoting effects of TSP2, though not to the same level as the effect of TSP2 in males.

These findings highlight an important and thus far overlooked sex specific regulation of synapse formation by astrocytes, and lay the groundwork for future research of sex specific synapse modulation by additional astrocyte secreted factors. Overall, the manuscript is well written and the data quality is excellent, and is presented clearly. However, several key points to strengthen the study’s conclusions and interpretation are missing and should be addressed:

Major points:

1. In Figures 1-2, the authors show that the astrocyte media from females is as potent to induce synapse formation in males as the male media. While this suggests, the levels of TSP2 in both sex media is the same, astrocytes secrete multiple factors, some with overlapping function (including other TSP family members), all of which could similarly induce changes in synapse number as analyzed by ICC. Furthermore, the diminished response of female neurons could also be due to differences in expression levels of the TSP2 receptor, a2d. Is there a difference in secreted TSP2 levels between males and females? Is there a difference in the expression levels of a2d between males and females?

2. The mechanistic link between TSP2-mediated synapse formation and estrogen is weakly supported. In females, the effect of TSP2 alone (Figure 4C) is very similar to that of TSP2+letrozole (authors should add the outcome of statistical analysis to the plot for all comparisons). In males, as also pointed out in Discussion, the results of letrozole treatment are contrary to the initial hypothesis. Still, disrupting estrogen synthesis clearly has an effect on TSP2 mediated synaptogenesis, however, is that disruption specific to the described sex-dependent modulation by TSP2, or is estrogen generally important for astrocyte-mediated synapse formation?

3. Is the inability of female neurons to respond to TSP2 restricted to early developmental period, when the estrogen levels are different (Figure 3A, DIV8-9)? If the neurons from both sexes are treated with TSP2 at DIV14-21, after the developmental differences in estrogen levels disappear, but still long before puberty related hormonal changes occur, would there still be sex-dependent difference in TSP2-induced synapse number?

Minor points:

1. Figures 1B and 1C show overlapping data of the increase in synapse number in neurons of each sex, treated by sex matched astrocyte media. It would make this results section clearer if they were combined to one panel, especially since the difference in the magnitude of the effect on synapse number between the males and females is only statistically significant for data presented in Figure 1C.

## Author Response

We thank the reviewers for their overall enthusiasm for our study and for their careful critiques, which we have now addressed with this revised version of the manuscript. We have performed additional experiments to better elucidate the role of estrogen in TSP-induced synaptogenesis, as well as intrinsic sex differences in TSP and its receptor α2δ-1. At the request of the editors, we have implemented the use of estimation statistics (where appropriate) in order to enhance transparency and emphasize the rigor of our statistical analyses. The manuscript text has been revised accordingly, with significant additions highlighted in yellow and deletions in red. We hope that you find this revised version acceptable for publication in eNeuro.

Below are our point-by-point responses to the reviewers’ comments (original comments in quotations).

Reviewer 1:

"In this study, the authors reveal a surprising sex difference in the regulation of synaptic connectivity by astrocyte-derived secreted factors. The authors find that synaptogenesis in female cortical neurons is less responsive to secreted factors from cocultured astrocytes or from astrocyte-conditioned medium.

Strikingly, treatment of male and female cortical neuron cultures with the astrocyte-derived secreted factor Thrombospondin-2 (TSP2) reveals that only male neurons increase their synapse density in response to TSP2. The authors then explore a potential contribution of sex hormones to these differences. They identify a small transient increase in estradiol levels in female neurons at DIV9, whereas glial levels are similar between male and female. Finally, the authors use letrozole, an inhibitor of the enzyme that converts testosterone to estrogen. This treatment slightly increases TSP2’s synaptogenic effect in female neurons. Surprisingly and unexpectedly, this treatment blocks TSP2- induced synaptogenesis in male neurons. Overall, this is an interesting study that emphasizes the need to take sex differences into account for studies investigating synaptogenic factors. The exact role of estrogen in TSP2-mediated synaptogenesis remains unclear.”

We thank the reviewer for their positive remarks concerning the importance and potential impact of this study.

"Specific comments:

The role of estrogen is unclear. I don’t expect the authors to fully elucidate the underlying mechanism, but additional insight is necessary.”

We apologize for our lack of clarity with regards to the role of estrogen in astrocyte-mediated synaptogenesis. Part of this stems from the fact that there is still much to uncover in terms of how estrogen signaling affects these critical developmental processes, which is a major focus of our ongoing work. Still, we felt that our current findings were novel and important enough to disseminate to the greater research community even in this relatively early stage of discovery. However, the reviewer’s point is well-taken. To accommodate this request, we have performed additional experiments to test the role of estradiol and aromatase on the expression of α2δ-1 (Figure 4B), as well as the synaptogenic consequences of manipulating estrogen signaling pathways via application of exogenous estradiol, an estrogen receptor (ER) antagonist, or an aromatase agonist (Figure 6). Interestingly, though we did not detect any sex differences or effects of estradiol/aromatase on α2δ-1 expression, we did gather further evidence that modulation of endogenous estrogen signaling (either stimulating or inhibiting) tended to interfere with TSP-induced synaptogenesis. In addition to this new data, we have also updated the discussion with further speculation as to estrogen’s involvement in astrocyte-mediated synaptic development.2

"1. There is a small difference in male vs female estradiol levels in neurons at DIV9 and blocking estrogen synthesis mildly promotes TSP2-mediated synaptogenesis in female neurons. Would TSP2 treatment of cortical neurons at an earlier stage in vitro (e.g. DIV2-7), when estradiol levels are equally low in male and female neurons, have a synaptogenic effect on female neurons?"

α2δ-1 is developmentally regulated and is not observed at appreciable levels in the cortex until after P5 (Risher et al., J Cell Bio 2018), which is why our treatment timeline (i.e. DIV7-13) was chosen to coincide with both the upregulation of α2δ-1 and the peak period of TSP expression and TSP-induced synaptogenesis in vivo (Christopherson et al., Cell 2005; Semple et al., Prog Neurobio 2013). However, some insight into this question was provided in an experiment we performed in response to one of

Reviewer 2’s comments regarding delayed TSP2 treatment (i.e. DIV13-19). Those results are discussed in the relevant section below.

"2. Are there differences in male vs female expression of the alpha2delta-1 TSP2 receptor (or additional

TSP receptors)? Potential differences in expression (mRNA and protein) might help explain sex-specific effects of TSP2.

3. Does estrogen affect alpha2delta-1 receptor levels? Does aromatase block affect alpha2delta-1 expression, especially in male neurons?"

To address comments 2 and 3, we performed Western blot analysis of α2δ-1 protein expression in cell lysates from purified neuron cultures of both sexes treated with exogenous estradiol (E2; 100 nM) or letrozole (100 nM) (Figure 4B). We found that α2δ-1 expression was not influenced by sex or

E2/letrozole, indicating that other factors are responsible for the sex-specific effects of TSP2.

"4. Some additional discussion on potential effects of aromatase blocking in male neurons would be helpful. Is aromatase equally expressed in male and female neurons? Would this block affect other processes in the cell? “

Though we did not directly test this in our study, the literature shows that a number of groups have previously investigated sex differences in aromatase expression throughout the CNS (Tabatadze, Sato and Woolley, PLoS One 2014; Bender et al., J Neurosci 2017; Fels, Casalena and Manfredi, Endocrin Diab

Metab 2020). Though the studies varied in terms of age as well as methodology (mRNA vs. protein), a common conclusion is that sex differences do exist in some brain regions but not others. Cortex and hippocampus, for example, are typically cited as areas that do not show significant sex differences in aromatase expression, yet other areas such as the preoptic area of the hypothalamus and the bed nucleus of the stria terminalis (BNST) present with strong sex biases. Since the list of CNS functions influenced by estrogen signaling is quite extensive (McCarthy, Physiol Rev 2008), it would stand to reason that blocking aromatase would have widespread consequences that go far beyond the synaptogenic mechanism that we are investigating here. However, there are precedents for sex-specific synaptic responses to letrozole in the hippocampus and basolateral amygdala, despite similar levels of aromatase expression (Vierk et al., J Neurosci 2012; Bender et al., J Neurosci 2017).

The reviewer’s comments also prompted us to think about aromatase signaling more broadly. We were thinking specifically about the ability of aromatase to catalyze the conversion of testosterone to estradiol, yet inhibiting this pathway would make more testosterone available for conversion to dihydrotestosterone (DHT) via 5α-reductase. DHT would then go on to stimulate androgen receptors, 3 which has its own effects on synaptic development (Brandt et al., Cereb Cortex 2019). Because we observed direct effects of estradiol and estrogen receptor activity on TSP-induced synapse formation (Figure 6), we felt that diving deeper into E2/ER vs. DHT/AR signaling would be beyond the scope of the current study. As such, we are planning a much more extensive investigation in the future but have updated the current manuscript’s Discussion with these additional speculations.

"5. It would be helpful to indicate how long cultures were treated with TSP2 in the legends of Figures 2 and 4. From the Methods it seems treatment was done on DIV7 and DIV10, and analysis on DIV13, but please specify for ease of viewing.”

We apologize for the confusion in the experimental design. The treatments were indeed performed on

DIV7 and DIV10, followed by fixation on DIV13. We have now included this information in the relevant figure legends.

Reviewer 2:

"In this manuscript the authors examine the sex differences in synapse regulation by astrocytes. The authors show that media collected from cultured astrocytes from either sex, induces formation of more synapses in males than in females. The authors then examine the effects of an astrocyte secreted synapse regulating factor Thrombospondin 2 (TSP2), and find that it selectively promotes synapse formation in males, providing possible explanation for the differences seen in neurons treated with the astrocyte culture media. To identify the underlying mechanism the authors analyzed changes in levels of the sex hormone estrogen, and observed transiently higher levels of estrogen in female neurons.

Treatment of female neurons with estrogen synthesis blocker letrozole, increased their response to synapse promoting effects of TSP2, though not to the same level as the effect of TSP2 in males.

These findings highlight an important and thus far overlooked sex specific regulation of synapse formation by astrocytes, and lay the groundwork for future research of sex specific synapse modulation by additional astrocyte secreted factors. Overall, the manuscript is well written and the data quality is excellent, and is presented clearly. However, several key points to strengthen the study’s conclusions and interpretation are missing and should be addressed:”

We thank the reviewer for their enthusiastic impressions regarding this work.

"Major points:

1. In Figures 1-2, the authors show that the astrocyte media from females is as potent to induce synapse formation in males as the male media. While this suggests, the levels of TSP2 in both sex media is the same, astrocytes secrete multiple factors, some with overlapping function (including other TSP family members), all of which could similarly induce changes in synapse number as analyzed by ICC.

Furthermore, the diminished response of female neurons could also be due to differences in expression levels of the TSP2 receptor, a2d. Is there a difference in secreted TSP2 levels between males and females? Is there a difference in the expression levels of a2d between males and females?"

While the levels of purified TSP2 in our treatments was identical between sexes (500 ng/mL for both), the reviewer is correct in noting that there is a possibility of different levels of secreted factors in astrocyte-conditioned media (ACM) between male and female astrocytes. To test this, we performed

Western blot analysis of secreted TSP2 protein levels from cultured astrocytes at DIV10 and did not observe any significant differences (Figure 4A). Additionally, we performed Western blot analysis of α2δ-4

1 protein expression in cell lysates from purified neuron cultures of both sexes and found no difference at DIV13, the final day of our standard treatment timeline (Figure 4B).

"2. The mechanistic link between TSP2-mediated synapse formation and estrogen is weakly supported.

In females, the effect of TSP2 alone (Figure 4C) is very similar to that of TSP2+letrozole (authors should add the outcome of statistical analysis to the plot for all comparisons). In males, as also pointed out in

Discussion, the results of letrozole treatment are contrary to the initial hypothesis. Still, disrupting estrogen synthesis clearly has an effect on TSP2 mediated synaptogenesis, however, is that disruption specific to the described sex-dependent modulation by TSP2, or is estrogen generally important for astrocyte-mediated synapse formation?"

This comment mirrors the one made by Reviewer 1 with regards to the weak support for the influence of estrogen on TSP2-mediated synaptogenesis. As stated previously, we had intended for this manuscript to introduce the potential paradigm shift of estrogen/sex hormones as regulators of astrocyte-mediated synaptic development, but we are aware now that we did not include enough evidence or speculation in our original submission. To correct that oversight, we have performed several additional experiments that we think strongly bolster our argument in favor of estrogen as a major mediator of TSP/astrocyte-induced synapse formation. In the first of these, we applied letrozole to male and female-derived neuron cultures that were being treated with either astrocyte inserts or ACM in order to determine whether inhibiting aromatase would more generally interfere with astrocyte mediated synaptogenic pathways (Figure 5E). In these treatments, we observed a consistent effect of letrozole decreasing synapse number compared to the astrocyte/ACM treatment (similar to that observed in male neurons treated with TSP2 + letrozole). In another new experiment, we chose an opposing strategy and sought to combine our TSP2 treatments with prostaglandin E2 (PGE2), an aromatase agonist, as well as two other factors involved in estrogen signaling: exogenous estradiol/E2 and ICI 182 780 (estrogen receptor alpha/beta antagonist) (Figure 6). Whereas inhibiting aromatase with letrozole mildly increased synapse number when combined with TSP2 in both male and female neurons, stimulating aromatase activity with PGE2 resulted in a net decrease in synapses with TSP2 treatment.

Furthermore, strongly manipulating estrogen signaling in either direction, either stimulating by adding exogenous E2 or inhibiting estrogen receptors with ICI 182 780, completely precluded the ability of TSP2 to promote synaptogenesis in either sex. While we are still far from a complete understanding of the mechanisms in play, we hope that the additional experiments (and accompanying discussion) have provided enough evidence for estrogen’s modulatory role in these critical pathways.

"3. Is the inability of female neurons to respond to TSP2 restricted to early developmental period, when the estrogen levels are different (Figure 3A, DIV8-9)? If the neurons from both sexes are treated with

TSP2 at DIV14-21, after the developmental differences in estrogen levels disappear, but still long before puberty related hormonal changes occur, would there still be sex-dependent difference in TSP2-induced synapse number?"

To address this possibility, we performed a delayed TSP2/letrozole treatment on our male and female derived neuron cultures: beginning with DIV13 (i.e. the day that we fix the neurons in our standard treatment timeline), treating again on DIV16, then fixing on DIV19. With this delayed timeline, we were no longer able to detect any TSP2-induced increases in synapse number in either sex (Figure 5D), supporting the existence of a sex-specific critical window for TSP2-induced synapse formation during the second postnatal week.5

"Minor points:

1. Figures 1B and 1C show overlapping data of the increase in synapse number in neurons of each sex, treated by sex matched astrocyte media. It would make this results section clearer if they were combined to one panel, especially since the difference in the magnitude of the effect on synapse number between the males and females is only statistically significant for data presented in Figure 1C.”

These panels actually represent two different experiments, with panel 1B showing the results of applying astrocyte inserts to the neuron culture wells (thereby allowing the continuous exchange of media between the two live cell types), and panel 1C showing the effects of timed treatments of previously purified ACM. Based on these differences, we feel that it would be better to keep the panels separate. However, since this presentation did result in confusion for the Reviewer, we have modified the figure legends to better reflect the differences in these experiments

## References

[B1] Abel KM, Drake R, Goldstein JM (2010) Sex differences in schizophrenia. Int Rev Psychiatry 22:417–428. 10.3109/09540261.2010.515205 21047156

[B2] Allen NJ, Eroglu C (2017) Cell biology of astrocyte-synapse interactions. Neuron 96:697–708. 10.1016/j.neuron.2017.09.056 29096081 PMC5687890

[B3] Allen NJ, Bennett ML, Foo LC, Wang GX, Chakraborty C, Smith SJ, Barres BA (2012) Astrocyte glypicans 4 and 6 promote formation of excitatory synapses via GluA1 AMPA receptors. Nature 486:410–414. 10.1038/nature11059 22722203 PMC3383085

[B4] Alonso-Nanclares L, Gonzalez-Soriano J, Rodriguez JR, DeFelipe J (2008) Gender differences in human cortical synaptic density. Proc Natl Acad Sci USA 105:14615–14619. 10.1073/pnas.0803652105 18779570 PMC2567215

[B5] Azcoitia I, Yague JG, Garcia-Segura LM (2011) Estradiol synthesis within the human brain. Neuroscience 191:139–147. 10.1016/j.neuroscience.2011.02.012 21320576

[B6] Barres BA (2008) The mystery and magic of glia: a perspective on their roles in health and disease. Neuron 60:430–440. 10.1016/j.neuron.2008.10.013 18995817

[B7] Bender RA, Zhou L, Vierk R, Brandt N, Keller A, Gee CE, Schäfer MK, Rune GM (2017) Sex-dependent regulation of aromatase-mediated synaptic plasticity in the basolateral amygdala. J Neurosci 37:1532–1545. 10.1523/JNEUROSCI.1532-16.2016 28028198 PMC6705682

[B8] Blanco-Suarez E, Liu TF, Kopelevich A, Allen NJ (2018) Astrocyte-secreted chordin-like 1 drives synapse maturation and limits plasticity by increasing synaptic GluA2 AMPA receptors. Neuron 100:1116–1132.e3. 10.1016/j.neuron.2018.09.043 30344043 PMC6382071

[B9] Brandt N, Rune GM (2020) Sex-dependency of oestrogen-induced structural synaptic plasticity: inhibition of aromatase versus application of estradiol in rodents. Eur J Neurosci 52:2548–2559. 31403726 10.1111/ejn.14541

[B10] Brandt N, Vierk R, Fester L, Anstötz M, Zhou L, Heilmann LF, Kind S, Steffen P, Rune GM (2020) Sex-specific difference of hippocampal synaptic plasticity in response to sex neurosteroids. Cereb Cortex 30:2627–2641. 10.1093/cercor/bhz265 31800024

[B11] Chaban VV, Lakhter AJ, Micevych P (2004) A membrane estrogen receptor mediates intracellular calcium release in astrocytes. Endocrinology 145:3788–3795. 10.1210/en.2004-0149 15131017

[B12] Christopherson KS, Ullian EM, Stokes CC, Mullowney CE, Hell JW, Agah A, Lawler J, Mosher DF, Bornstein P, Barres BA (2005) Thrombospondins are astrocyte-secreted proteins that promote CNS synaptogenesis. Cell 120:421–433. 10.1016/j.cell.2004.12.020 15707899

[B13] Diniz LP, Matias IC, Garcia MN, Gomes FC (2014) Astrocytic control of neural circuit formation: highlights on TGF-beta signaling. Neurochem Int 78:18–27. 10.1016/j.neuint.2014.07.008 25125369

[B14] Eroglu C, Allen NJ, Susman MW, O’Rourke NA, Park CY, Ozkan E, Chakraborty C, Mulinyawe SB, Annis DS, Huberman AD, Green EM, Lawler J, Dolmetsch R, Garcia KC, Smith SJ, Luo ZD, Rosenthal A, Mosher DF, Barres BA (2009) Gabapentin receptor alpha2delta-1 is a neuronal thrombospondin receptor responsible for excitatory CNS synaptogenesis. Cell 139:380–392. 10.1016/j.cell.2009.09.025 19818485 PMC2791798

[B15] Farhy-Tselnicker I, Allen NJ (2018) Astrocytes, neurons, synapses: a tripartite view on cortical circuit development. Neural Dev 13:7. 10.1186/s13064-018-0104-y 29712572 PMC5928581

[B16] Farhy-Tselnicker I, van Casteren ACM, Lee A, Chang VT, Aricescu AR, Allen NJ (2017) Astrocyte-secreted glypican 4 regulates release of neuronal pentraxin 1 from axons to induce functional synapse formation. Neuron 96:428–445.e13. 10.1016/j.neuron.2017.09.053 29024665 PMC5663462

[B17] Fels JA, Casalena GA, Manfredi G (2021) Sex and oestrogen receptor beta have modest effects on gene expression in the mouse brain posterior cortex. Endocrinol Diabetes Metab 4:e00191. 10.1002/edm2.191 33532622 PMC7831211

[B18] Genazzani AR, Pluchino N, Luisi S, Luisi M (2007) Estrogen, cognition and female ageing. Hum Reprod Update 13:175–187. 10.1093/humupd/dml042 17135285

[B19] Gillies GE, McArthur S (2010) Estrogen actions in the brain and the basis for differential action in men and women: a case for sex-specific medicines. Pharmacol Rev 62:155–198. 10.1124/pr.109.002071 20392807 PMC2879914

[B20] Grkovic I, Mitrovic N (2020) Estradiol induces synaptic rearrangements. Vitam Horm 114:233–256. 32723546 10.1016/bs.vh.2020.04.006

[B21] Hanamsagar R, Bilbo SD (2016) Sex differences in neurodevelopmental and neurodegenerative disorders: focus on microglial function and neuroinflammation during development. J Steroid Biochem Mol Biol 160:127–133. 10.1016/j.jsbmb.2015.09.039 26435451 PMC4829467

[B22] Harrison PJ, Tunbridge EM, Dolphin AC, Hall J (2020) Voltage-gated calcium channel blockers for psychiatric disorders: genomic reappraisal. Br J Psychiatry 216:250–253. 31230606 10.1192/bjp.2019.157 PMC7557861

[B23] Ho J, Tumkaya T, Aryal S, Choi H, Claridge-Chang A (2019) Moving beyond P values: data analysis with estimation graphics. Nat Methods 16:565–566. 10.1038/s41592-019-0470-3 31217592

[B24] Ho TC, Colich NL, Sisk LM, Oskirko K, Jo B, Gotlib IH (2020) Sex differences in the effects of gonadal hormones on white matter microstructure development in adolescence. Dev Cogn Neurosci 42:100773. 10.1016/j.dcn.2020.100773 32452463 PMC7058897

[B25] Hojo Y, Hattori TA, Enami T, Furukawa A, Suzuki K, Ishii HT, Mukai H, Morrison JH, Janssen WG, Kominami S, Harada N, Kimoto T, Kawato S (2004) Adult male rat hippocampus synthesizes estradiol from pregnenolone by cytochromes P45017alpha and P450 aromatase localized in neurons. Proc Natl Acad Sci USA 101:865–870. 10.1073/pnas.2630225100 14694190 PMC321772

[B26] Hösli E, Hösli L (1999) Cellular localization of estrogen receptors on neurones in various regions of cultured rat CNS: coexistence with cholinergic and galanin receptors. Int J Dev Neurosci 17:317–330. 10479067 10.1016/s0736-5748(99)00038-6

[B27] Hösli E, Jurasin K, Rühl W, Luthy R, Hösli L (2001) Colocalization of androgen, estrogen and cholinergic receptors on cultured astrocytes of rat central nervous system. Int J Dev Neurosci 19:11–19. 10.1016/S0736-5748(00)00082-4 11226751

[B28] Hughes EG, Elmariah SB, Balice-Gordon RJ (2010) Astrocyte secreted proteins selectively increase hippocampal GABAergic axon length, branching, and synaptogenesis. Mol Cell Neurosci 43:136–145. 10.1016/j.mcn.2009.10.004 19850128 PMC2818511

[B29] Iossifov I, O’Roak BJ, Sanders SJ, Ronemus M, Krumm N, Levy D, Stessman HA, Witherspoon KT, Vives L, Patterson KE, Smith JD, Paeper B, Nickerson DA, Dea J, Dong S, Gonzalez LE, Mandell JD, Mane SM, Murtha MT, Sullivan CA, et al. (2014) The contribution of de novo coding mutations to autism spectrum disorder. Nature 515:216–221. 10.1038/nature13908 25363768 PMC4313871

[B30] Ippolito DM, Eroglu C (2010) Quantifying synapses: an immunocytochemistry-based assay to quantify synapse number. J Vis Exp. Advance online publication. Retrieved Nov 16, 2010. doi: 10.3791/2270. PMC3159596 21113117

[B31] Konkle AT, McCarthy MM (2011) Developmental time course of estradiol, testosterone, and dihydrotestosterone levels in discrete regions of male and female rat brain. Endocrinology 152:223–235. 10.1210/en.2010-0607 21068160 PMC3033055

[B32] Kramár EA, Chen LY, Brandon NJ, Rex CS, Liu F, Gall CM, Lynch G (2009) Cytoskeletal changes underlie estrogen’s acute effects on synaptic transmission and plasticity. J Neurosci 29:12982–12993. 10.1523/JNEUROSCI.3059-09.2009 19828812 PMC2806054

[B33] Kretz O, Fester L, Wehrenberg U, Zhou L, Brauckmann S, Zhao S, Prange-Kiel J, Naumann T, Jarry H, Frotscher M, Rune GM (2004) Hippocampal synapses depend on hippocampal estrogen synthesis. J Neurosci 24:5913–5921. 10.1523/JNEUROSCI.5186-03.2004 15229239 PMC6729232

[B34] Kucukdereli H, Allen NJ, Lee AT, Feng A, Ozlu MI, Conatser LM, Chakraborty C, Workman G, Weaver M, Sage EH, Barres BA, Eroglu C (2011) Control of excitatory CNS synaptogenesis by astrocyte-secreted proteins Hevin and SPARC. Proc Natl Acad Sci USA 108:E440–E449. 10.1073/pnas.1104977108 21788491 PMC3156217

[B35] Leranth C, Petnehazy O, MacLusky NJ (2003) Gonadal hormones affect spine synaptic density in the CA1 hippocampal subfield of male rats. J Neurosci 23:1588–1592. 10.1523/JNEUROSCI.23-05-01588.2003 12629162 PMC6741990

[B36] Lu Y, Sareddy GR, Wang J, Wang R, Li Y, Dong Y, Zhang Q, Liu J, O’Connor JC, Xu J, Vadlamudi RK, Brann DW (2019) Neuron-derived estrogen regulates synaptic plasticity and memory. J Neurosci 39:2792–2809. 10.1523/JNEUROSCI.1970-18.2019 30728170 PMC6462452

[B37] McCarthy MM (2008) Estradiol and the developing brain. Physiol Rev 88:91–124. 10.1152/physrev.00010.2007 18195084 PMC2754262

[B38] McCarthy MM, Todd BJ, Amateau SK (2003) Estradiol modulation of astrocytes and the establishment of sex differences in the brain. Ann NY Acad Sci 1007:283–297. 10.1196/annals.1286.027 14993061

[B39] McCarthy MM, Arnold AP, Ball GF, Blaustein JD, De Vries GJ (2012) Sex differences in the brain: the not so inconvenient truth. J Neurosci 32:2241–2247. 10.1523/JNEUROSCI.5372-11.2012 22396398 PMC3295598

[B40] Mukai H, Tsurugizawa T, Murakami G, Kominami S, Ishii H, Ogiue-Ikeda M, Takata N, Tanabe N, Furukawa A, Hojo Y, Ooishi Y, Morrison JH, Janssen WG, Rose JA, Chambon P, Kato S, Izumi S, Yamazaki T, Kimoto T, Kawato S (2007) Rapid modulation of long-term depression and spinogenesis via synaptic estrogen receptors in hippocampal principal neurons. J Neurochem 100:950–967. 10.1111/j.1471-4159.2006.04264.x 17266735

[B41] Penzes P, Cahill ME, Jones KA, VanLeeuwen JE, Woolfrey KM (2011) Dendritic spine pathology in neuropsychiatric disorders. Nat Neurosci 14:285–293. 10.1038/nn.2741 21346746 PMC3530413

[B42] Pfaff DW, Joëls M (2017) Hormones, brain, and behavior. Amsterdam; Boston: Elsevier/Academic Press.

[B43] Prange-Kiel J, Wehrenberg U, Jarry H, Rune GM (2003) Para/autocrine regulation of estrogen receptors in hippocampal neurons. Hippocampus 13:226–234. 10.1002/hipo.10075 12699330

[B44] Risher WC, Eroglu C (2012) Thrombospondins as key regulators of synaptogenesis in the central nervous system. Matrix Biol 31:170–177. 10.1016/j.matbio.2012.01.004 22285841 PMC3961754

[B45] Risher WC, Patel S, Kim IH, Uezu A, Bhagat S, Wilton DK, Pilaz LJ, Singh Alvarado J, Calhan OY, Silver DL, Stevens B, Calakos N, Soderling SH, Eroglu C (2014) Astrocytes refine cortical connectivity at dendritic spines. Elife 3:e04047. 10.7554/eLife.04047 PMC4286724 25517933

[B46] Risher WC, Kim N, Koh S, Choi JE, Mitev P, Spence EF, Pilaz LJ, Wang D, Feng G, Silver DL, Soderling SH, Yin HH, Eroglu C (2018) Thrombospondin receptor α2δ-1 promotes synaptogenesis and spinogenesis via postsynaptic Rac1. J Cell Biol 217:3747–3765. 10.1083/jcb.201802057 30054448 PMC6168259

[B47] Semple BD, Blomgren K, Gimlin K, Ferriero DM, Noble-Haeusslein LJ (2013) Brain development in rodents and humans: identifying benchmarks of maturation and vulnerability to injury across species. Prog Neurobiol 106–107:1–16. 10.1016/j.pneurobio.2013.04.001 23583307 PMC3737272

[B48] Srivastava DP, Penzes P (2011) Rapid estradiol modulation of neuronal connectivity and its implications for disease. Front Endocrinol (Lausanne) 2:77. 10.3389/fendo.2011.00077 22654827 PMC3356153

[B49] Tabatadze N, Sato SM, Woolley CS (2014) Quantitative analysis of long-form aromatase mRNA in the male and female rat brain. PLoS One 9:e100628. 10.1371/journal.pone.0100628 25036039 PMC4103800

[B50] Vierk R, Glassmeier G, Zhou L, Brandt N, Fester L, Dudzinski D, Wilkars W, Bender RA, Lewerenz M, Gloger S, Graser L, Schwarz J, Rune GM (2012) Aromatase inhibition abolishes LTP generation in female but not in male mice. J Neurosci 32:8116–8126. 10.1523/JNEUROSCI.5319-11.2012 22699893 PMC6703647

[B51] Weisz J, Ward IL (1980) Plasma testosterone and progesterone titers of pregnant rats, their male and female fetuses, and neonatal offspring. Endocrinology 106:306–316. 10.1210/endo-106-1-306 7349961

[B52] Werling DM, Geschwind DH (2013) Sex differences in autism spectrum disorders. Curr Opin Neurol 26:146–153. 10.1097/WCO.0b013e32835ee548 23406909 PMC4164392

[B53] Winzeler A, Wang JT (2013) Purification and culture of retinal ganglion cells from rodents. Cold Spring Harb Protoc 2013:643–652. 10.1101/pdb.prot074906 23818667

[B54] Woolley CS, McEwen BS (1992) Estradiol mediates fluctuation in hippocampal synapse density during the estrous cycle in the adult rat. J Neurosci 12:2549–2554. 10.1523/JNEUROSCI.12-07-02549.1992 1613547 PMC6575846

[B55] Zhao J, Bian C, Liu M, Zhao Y, Sun T, Xing F, Zhang J (2018) Orchiectomy and letrozole differentially regulate synaptic plasticity and spatial memory in a manner that is mediated by SRC-1 in the hippocampus of male mice. J Steroid Biochem Mol Biol 178:354–368. 10.1016/j.jsbmb.2018.02.007 29452160

